# Whole genome sequencing, molecular typing and *in vivo* virulence of OXA-48-producing *Escherichia coli* isolates including ST131 *H*30-Rx, *H*22 and *H*41 subclones

**DOI:** 10.1038/s41598-017-12015-0

**Published:** 2017-09-21

**Authors:** María de Toro, Javier Fernández, Vanesa García, Azucena Mora, Jorge Blanco, Fernando de la Cruz, M. Rosario Rodicio

**Affiliations:** 1 Plataforma de Genómica y Bioinformática, Centro de Investigación Biomédica de La Rioja (CIBIR), Logroño, Spain; 2 Departamento de Biología Funcional, Área de Microbiología, Universidad de Oviedo (UO), Oviedo, Spain; 3 Servicio de Microbiología, Hospital Universitario Central de Asturias (HUCA), Oviedo, Spain; 4 Laboratorio de Referencia de Escherichia coli (LREC), Departamento de Microbioloxía e Parasitoloxía, Facultade de Veterinaria, Universidade de Santiago de Compostela (USC), Lugo, Spain; 5 Departamento de Biología Molecular and Instituto de Biomedicina y Biotecnología de Cantabria (IBBTEC), Universidad de Cantabria-CSIC, Santander, Spain

## Abstract

Carbapenem-resistant *Enterobacteriaceae*, including the increasingly reported OXA-48 *Escherichia coli* producers, are an emerging public health threat worldwide. Due to their alarming detection in our healthcare setting and their possible presence in the community, seven OXA-48-producing, extraintestinal pathogenic *E*. *coli* were analysed by whole genome sequencing as well as conventional tools, and tested for *in vivo* virulence. As a result, five *E*. *coli* OXA-48-producing subclones were detected (O25:H4-ST131/PST43-*fimH*30-virotype E; O25:H4-ST131/PST9-*fimH*22-virotype D5, O16:H5-ST131/PST506-*fimH*41; O25:H5-ST83/PST207 and O9:H25-ST58/PST24). Four ST131 and one ST83 isolates satisfied the ExPEC status, and all except the O16:H5 ST131 isolate were UPEC. All isolates exhibited local inflammatory response with extensive subcutaneous necrosis but low lethality when tested in a mouse sepsis model. The *bla*_OXA-48_ gene was located in MOB_P131_/IncL plasmids (four isolates) or within the chromosome (three ST131 *H*30-Rx isolates), carried by Tn*1999*-like elements. All, except the ST83 isolate, were multidrug-resistant, with additional plasmids acting as vehicles for the spread of various resistance genes. This is the first study to analyse the whole genome sequences of *bla*_OXA-48_-positive ST131, ST58 and ST83 *E*. *coli* isolates in conjunction with experimental data, and to evaluate the *in vivo* virulence of *bla*_OXA-48_ isolates, which pose an important challenge to patient management.

## Introduction

*Escherichia coli* is a common member of the intestine microbiota of warm-blooded vertebrates including humans. It can cause a range of conditions, from diarrheagenic diseases to extraintestinal infections^1^. Extraintestinal pathogenic *E*. *coli* (ExPEC) lineages are involved in the latter. Within them, the clonal group ST131 and its *H*30-R and *H*30-Rx subclones (clades C1 and C2), are associated with antimicrobial resistance and have successfully spread, creating a global epidemic of multidrug-resistant (MDR) *E*. *coli* infections^1–3^. Treatment of MDR *E*. *coli* infections has become a serious clinical issue, with carbapenems being one of the last therapeutic options^4^. Unfortunately, carbapenemase production is increasingly been reported in *E*. *coli*, as well as in other *Enterobacteriaceae* ^4,5^. The *bla*_OXA-48_ gene, which encodes the OXA-48 carbapenem-hydrolysing class D β-lactamase, has been detected in several *E*. *coli* clonal groups, including ST131^6,7^. The *bla*_OXA-48_ gene is usually located on self-transferable plasmids belonging to the IncL/M incompatibility group (recently re-classified into IncL and IncM^8^), largely responsible for its dissemination between members of the *Entebacteriaceae* ^5,8^. Occasionally, *bla*_OXA-48_ has also been found in the *E*. *coli* chromosome^9,10^. In both cases, plasmids and chromosomes, the gene is carried by Tn*1999*-like composite transposons, designated Tn*1999*.*1* to Tn*1999*.*4* ^9,11–14^.

Due to the alarming emergence of OXA-48-producing *E*. *coli* in our healthcare area, this study aimed to (i) analyse the genomic diversity of the isolates, using a combination of whole genome sequencing and molecular typing; (ii) investigate the role of plasmids in the dissemination of resistance and virulence genes, and (iii) assess the lethality of the isolates using a mouse sepsis model. For this purpose, seven OXA-48-producing *E*. *coli* recovered between 2012 and 2015 from clinical samples in three different settings within a Spanish city (Oviedo), were selected (Table 1).

**Table 1:** Origin, resistance properties and molecular typing of OXA-48 β-Lactamase *Escherichia coli* isolates from a Spanish hospital.

Isolate	Year of isolation	Patient Sex^a^/age^b^	Origin^c^	Sample	Resistance phenotype^d^/genes^e^	ERT/IPM/MER MIC	Serotype	MLST^f^	*fimH* allele	Virotype^g^	Phylogroup
*Ec*-HUCA 1^h^	2012	F/46	GSU	Surgical wound	AMP, AMC, ETP, IPM^I^, ERY^i^, NAL, TET/*bla*_TEM-1b_, *bla*_OXA-48,_ *mph* (A), *gyrA*-S83L, *tet* (B)	2/2/0.19	O16:H5	ST131/PST506	*H*41	nt	B2
*Ec*-HUCA 2^h^	2012	M/57	ICU	Surgical wound	AMP, AMC, CTX, ETP, IMP^I^, [AMK^I^, GEN, TOB], KAN, STR, ERY^i^, SUL, TMP, SXT,TET/*bla*_TEM-1b_, *bla*_OXA-30_, *bla*_CTX-M-15_, *bla*_OXA-48,_ *aph* (3′)-Ia, *aadA1*, *strB*, *mph* (A), *sul1*, *dfrA5*, *tet* (A)	2/2/0.125	O9:H25	ST58/PST24	*H*27	na	B1
*Ec*-HUCA 3^h^	2012	M/68	RU	Urine	AMP, AMC, ETP, IMP^I^/*ampC2*, *ampH*, *bla*_OXA-48_	2/2/0.38	O25:H5	ST83/PST207	*H*21	na	B2
*Ec*-HUCA 4	2014	M/45	ICU	Surgical wound	AMP, AMC, FOX, ETP, IPM^I^, MER, CHL, NAL, SUL, TMP, SXT, TET/*bla*_TEM-1c_, *bla*_OXA-48,_ *cmlA1*, *gyrA*-S83L, *sul3*, *tet* (A)	4/2/3	O25:H4	ST131/PST9	*H*22	D5	B2
*Ec*-HUCA 5	2015	F/79	PCC	Urine	AMP, AMC, FOX, CTX, ETP, NAL, CIP, SXT/*bla*_CTX-M-15_, *bla*_OXA-48_, *gyrA*-S83L + D87N, *parC*-S80I + E84V, *folA*-W30R	2/0.5/0.38	O25:H4	ST131/PST43	*H*30	E	B2
*Ec*-HUCA 6	2015	M/80	EU	Urine	AMP, AMC, FOX, CTX, ETP, CHL, NAL, CIP, SXT, TMP/*bla*_CTX-M-15_, *bla*_OXA-48_, *gyrA*-S83L + D87N, *parC*-S80I + E84V, *qnrS1*, *dfrA14*	2/0.5/0.38	O25:H4	ST131/PST43	*H*30	E	B2
*Ec*-HUCA 7	2015	F/86	GU	Urine	AMP, AMC, FOX, CTX, ETP, CHL, NAL, CIP, SXT, TMP/*bla*_CTX-M-15_, *bla*_OXA-48_, *gyrA*-S83L + D87N, *parC*-S80I + E84V; *parE*-I529L, *qnrS1*, *dfrA14*	2/0.5/0.38	O25:H4	ST131/PST43	*H*30	E	B2

^a^F, female; M, male. ^b^years old. ^c^GSU, general surgery unit-HUCA; ICU, intensive care unit-HUCA; RU, reanimation unit-HUCA; PCC, primary-care center; EU, emergency unit-HUCA; GU, geriatric unit of a long-term care facility; HUCA, Hospital Universitario Central de Asturias. ^d^AMP, ampicillin; AMC, amoxicillin-clavulanic acid; FOX, cefoxitin; CTX, cefotaxime; ETP, ertapenem; IMP, imipenem; MER, meropenem; CHL, chloramphenicol, AMK, amikacin; GEN, gentamicin; KAN, kanamycin; TOB, tobramycin; STR, streptomycin, ERY, erythromycin; NAL, nalidixic acid; CIP, ciprofloxacin; SUL, sulfonamides; TMP, trimethoprim; SXT, trimethoprim-sulfamethoxazole; TET, tetracycline; I, intermediate resistance. ^e^All resistance genes were *in silico* determined; *bla*_CTX-M-15_ and *bla*_OXA-48_ were also experimentally detected by PCR amplification. Plasmid genes are underlined (see Table 2 for details). ^f^ST, sequence type according to Achtman; PST, sequence type according to the Pasteur Institute. ^g^The virotype was determined by PCR based on the presence or absence of 13 virulence genes^33^; nt, not typeable; na, not applicable. ^h^Isolates partially characterized in a previous study^15^. ^i^MIC of erythromycin: 256 µg/ml, higher than that obtained for isolates lacking *mph*(A): 128 µg/ml (*Ec*-HUCA 3, *Ec*-HUCA 5, *Ec*-HUCA 6, *Ec*-HUCA 7) or 64 µg/ml (*Ec*-HUCA 4).

## Results and Discussion

### Resistance properties and molecular typing of the isolates

As shown previously for isolates *Ec*-HUCA 1 to 3^15^, *Ec*-HUCA 4 to 7 showed increased MICs to ertapenem. Fortunately, the level of resistance to other carbapenems was low. In fact, all isolates except *Ec*-HUCA-4 were susceptible to meropenem, so this drug could have been included in the therapy regimen for the treatment of the affected patients (Table 1). In addition, all isolates were positive in the modified Hodge and the Carba NP tests, and contained the *bla*_OXA-48_ gene (Table 1). In *Ec*-HUCA 1 to 4 the *bla*_OXA-48_ gene was carried by conjugative plasmids of about 60 Kb, experimentally assigned to the IncL group. The *bla*_OXA-48_ gene was chromosomally located in *Ec*-HUCA 5 to 7. All isolates except *Ec*-HUCA 3 were also resistant to other antimicrobial agents, including fluoroquinolones, aminoglycosides, trimethoprim-sulfamethoxazole and broad spectrum cephalosporins (Table 1). The latter resistance was detected in four isolates (*Ec*-HUCA 2, 5, 6 and 7), which tested positive for *bla*_CTX-M-15_ by PCR amplification and sequencing.

Five out of the seven isolates were identified as ST131 by the Achtman scheme^16^ and further differentiated into PST43 (*Ec*-HUCA 5 to 7), PST9 (*Ec*-HUCA 4) and PST506 (*Ec*-HUCA 1) according to the Pasteur Institute scheme^17^ (Table 1). All ST131 isolates belonged to serotype O25:H4, except *Ec*-HUCA 1 which was O16:H5. The three O25:H4-ST131/PST43 isolates carried *fimH*30, were resistant to fluoroquinolones and positive for *bla*_CTX-M-15_, thus belonged to subclone *H*30-Rx. The remaining two isolates (*Ec*-HUCA 2 and 3) showed different serotypes (O9:H25 and O25:H5), STs (ST58 and ST83), PSTs (PST24 and PST207) and *fimH* alleles (*H*27 and *H*21) (Table 1). ST58 has been identified in isolates of different origins (human, animal and environment), and was frequently associated with CTX-M production, particularly CTX-M-1^18,19^. However, besides *Ec*-HUCA-2, only one other ST58 OXA-48-producer has been reported^20^. In contrast to ST131 and ST58, little information exists on ST83, which was previously found in uropathogenic *E*. *coli* from cats^21^. In addition, a query of the Enterobase database (version 6^th^ May 2017) identified 22 ST83 entries from different sources (human, companion animals, wildlife and environment) and geographical regions. The presence of *bla*_OXA-48_ in the otherwise susceptible *Ec*-HUCA 3, demands further surveillance of the ST83 lineage. By PCR, the ST131 and ST83 isolates were assigned to phylogroup B2, while the ST58 isolate belonged to phylogroup B1.

The isolates were also analysed by *Xba*I-PFGE electrophoresis. The dendrogram generated from comparison of the macrorestriction profiles was consistent with the genetic diversity of the isolates. Thus, only the three ST131/PST43 *H*30-Rx isolates of virotype E clustered with similarity ≥85%. The remaining two ST131 isolates (*Ec*-HUCA 1 and 4) presented 76.7% and 65% similarity with that cluster (Supplementary Figure S1). Although the high diversity of the ST131 clonal group has been widely reported^3,22^, it was unexpected for this specific group of five ST131 OXA-48-producing isolates, since they were obtained in the same health area over a relatively short period of time.

### Sequencing of OXA-48-producing isolates and plasmid reconstruction

The draft genomes of the seven *E*. *coli* isolates yielded 22 to 135 contigs larger than 1 Kb, with assembly sizes ranging from 4.9 Mb (*Ec*-HUCA 3) to 5.4 Mb (*Ec*-HUCA 4); (average of 5.2 ± 0.137 Mb). This is the first time that the genomes of OXA-48-producing ST131 *E. coli* isolates of subclones *H*30-Rx, *H*22 and *H*41, ST58 and ST83 were sequenced. *In silico* determinations of serotypes, STs/PSTs and *fimH* alleles fully agreed with experimental data.

In order to establish the plasmid content of the seven *E*. *coli* genomes, the PLACNET protocol^23^ was used for plasmid reconstruction (Supplementary Figure S2). As shown in Table 2, plasmids were found in all isolates in numbers ranging from one (*Ec*-HUCA 5) to seven (*Ec*-HUCA 4). In the genomes of *Ec*-HUCA 2 and 4, PLACNET identified two plasmids that could not be separated and accounted for a total of 348 Kb [IncF (F2/51/40:A-:B1)] and 253 Kb [IncF (F2:A-:B1) plus IncI1 (ST48)], respectively. Plasmid extraction followed by visualization on agarose gels resolved two plasmids of ca. 140 and 110 Kb in *Ec*-HUCA 4, a single plasmid band of 150 Kb in *Ec*-HUCA 2 (maybe two plasmids of similar sizes), and fully confirmed the existence of all other plasmids reconstructed by PLACNET (Supplementary Figure S3).

**Table 2:** Plasmid content of OXA-48-producing *Escherichia coli* isolates and location of resistance and virulence genes.

Isolate	Plasmids Chromosome	Size (bp)	Contigs	Relaxase protein^a,e^	Replication rotein^a,e^	Inc group (pMLST)^b,e^	Resistance genes^c,e^	Virulence genes^d,e^
*Ec-*HUCA 1	p*Ec*-HUCA 1_1	131330	26	MOB_F12_	RepFII, RepFIB	IncF (F29:A-:B10)	*bla*_TEM-1b_, *mph*(A), *tet*(B)	*senB*, *finO*, *traT*
	p*Ec*-HUCA 1_2	61395	6	MOB_P131_	IncFII_RepA_superfamily (pfam02387)	IncL/M	*bla*_OXA-48_	nd
	p*Ec*-HUCA 1_3	40230	6	MOB_F11_	Rep3_superfamily (pfam01051)	IncN (ST9)	nd	nd
	p*Ec*-HUCA 1_4	34530	4	MOB_P3_	Rep3_superfamily (pfam01051)	IncX1	nd	nd
	ICE_*Ec*-HUCA 1	201132 ^f^	1	MOB_H2_	nd	nd	nd	nd
	Chr_*Ec-*HUCA 1	4903773	62	na	na	na	*gyrA*-S83L	*gad*, *fimABCDEFGHI*, *ibeBC*, *iha*, *pic*, *sfaX*, *usp*, *matB*, *betA*, *irp2*, *sitC*, *fyuA/psn*
*Ec-*HUCA 2	p*Ec-*HUCA 2_1 + 2	347908	346	MOB_F12_	RepFII (x3), RepFIB	IncF (F2/51/40:A:B1)	*bla*_TEM-1b_, *bla*_OXA-30_, *bla*_CTX-M-15_, *aadA1*, *strB*, *aph*(3’)-Ia, *mph*(A), *sul1*, *dfrA5*, *tet*(A)	*iroN*, *mchF*, *ompT*, *sitC*, *cba*, *hlyF*, *iroBCDE*, *iss*, *iucABCD*, *iutA*, *etsABC*, *finO*, *traT*, *cvaC*
	p*Ec-*HUCA 2_3	61304	6	MOB_P131_	IncFII_RepA_superfamily (pfam02387)	IncL/M	*bla*_OXA-48_	nd
	p*Ec-*HUCA 2_4	6187	1*	MOB_Q12_	Replicase (pfam03090) + priCT1 (pfam 08708)	nd	nd	*celB*
	p*Ec-*HUCA 2_5	3003	1*	MOB_P51_	RNAI-II replication system	ColE-like	nd	nd
	Chr_*Ec*-HUCA 2	4741812	134	na	na	na	nd	*gad*, *lpfA*, *fimABCDEFGHI*, *ibeBC*, *matB*, *betA*, *irp2*, *fyuA/psn*, *iss*
Ec-HUCA 3	p*Ec*-HUCA 3_1	111201	1*	no-MOB	nd	nd	nd	nd
	p*Ec*-HUCA 3_2	61879	6	MOB_P131_	IncFII_RepA_superfamily (pfam02387)	IncL/M	*bla*_OXA-48_	nd
	Chr_*Ec*-HUCA 3	4747878	64	na	na	na	*ampC2*, *ampH*	*gad*, *pic*, *fimABCDEFGHI*, *ibeABC*, *sfaX*, *usp*, *matB*, *betA*, *irp2*, *sitC*, *fyuA/psn*, *vat*, *papBCDFHJK*
*Ec-*HUCA 4	p*Ec*-HUCA 4_1 + 2	253046	24	MOB_F12_ + MOB_P12_	RepFII, RepFIB, IncFII_RepA_superfamily (pfam02387)	IncF (F2:A-:B1) + IncI1 (ST48)	*bla*_TEM-1c_, *cmlA1*, *tet*(A), *sul3*	*iroN*, *mchF*, *ompT*, *sitC*, *hlyF*, *iroBCDE*, *iss*, *iucABCD*, *iutA*, *etsABC*, *finO*, *traT*, *cvaC*
	p*Ec*-HUCA 4_3	62423	5	MOB_P131_	IncFII_RepA_superfamily (pfam02387)	IncL/M	*bla*_OXA-48_	nd
	p*Ec*-HUCA 4_4	33543	1*	MOB_P3_	Rep3_superfamily (pfam01051)	IncX4	nd	nd
	p*Ec*-HUCA 4_5	6544	1	MOB_P51_	RNAI-II replication system	ColE-like	nd	nd
	p*Ec*-HUCA 4_6	4502	1	MOB_P51_	RNAI-II replication system	ColE-like	nd	nd
	p*Ec*-HUCA 4_7	1546	1*	no-MOB	RepA_HTH36_pfam13730	nd	nd	nd
	Chr_*Ec*-HUCA 4	4996329	48	na	na	na	*gyrA*-S83L	*gad*, *mchF*, *iss*, *cnf1*, *pic*, *fimABCDEFGHI*, *ibeABC*, *iha*, *sfaX*, *usp*, *matB*, *betA*, *irp2*, *fyuA/psn*, *papBCDFHJK*, *hlyABCD*
*Ec-*HUCA 5	p*Ec*-HUCA 5_1	5251	1*	MOB_P51_	RNAI-II replication system	ColE-like	nd	nd
	Chr_*Ec*-HUCA 5	5187005	184	na	na	na	*bla*_OXA-48,_ *bla*_CTX-M-15_, *gyrA*-S83L + D87N; *parC*-S80I + E84V; *parE*-I529L, *folA*-W30R	*gad*, *cnf1*, *pic*, *fimABCDEFGHI*, *ibeBC*, *iha*, *usp*, *matB*, *betA*, *irp2*, *sitC*, *fyuA/psn*, *sat*, *papBCDFGHJK*, *cnf1*, *hlyABCD*, *iss*, *iucABCD*, *iutA*
*Ec-*HUCA 6	p*Ec*-HUCA 6_1	45832	14	MOB_F11_	Rep3_superfamily (pfam01051)	IncN (ST7)	*qnrS1*, *dfrA14*	
	p*Ec*-HUCA 6_2	5251	1*	MOB_P51_	RNAI-II replication system	ColE-like	nd	nd
	Chr_*Ec*-HUCA 6	5218580	123	na	na	na	*bla*_OXA-48_, *bla*_CTX-M-15_, *gyrA*-S83L + D87N; *parC*-S80I + E84V; *parE*-I529L	*gad*, *cnf1*, *pic*, *fimABCDEFGHI*, *ibeBC*, *iha*, *usp*, *matB*, *betA*, *irp2*, *sitC*, *fyuA/psn*, *sat*, *papBCDEFGHJK*, *cnf1*, *hlyABCD*, *iss*, *iucABCD*, *iutA*
*Ec-*HUCA 7	p*Ec*-HUCA 7_1	48671	14	MOB_F11_	Rep3_superfamily (pfam01051)	IncN (ST7)	*bla*_CTX-M-15_, *qnrS1*, *dfrA14*	nd
	p*Ec*-HUCA 7_2	5251	1*	MOB_P51_	RNAI-II replication system	ColE-like	nd	nd
	Chr_*Ec*HUCA 7	5186594	125	na	na	na	*bla*_OXA-48_, *gyrA*-S83L + D87N; *parC*-S80I + E84V; *parE*-I529L	*gad*, *cnf1*, *pic*, *fimABCDEFGHI*, *ibeBC*, *iha*, *usp*, *matB*, *betA*, *irp2*, *sitC*, *fyuA/psn*, *sat*, *papBCDEFGHJK*, *cnf1*, *hlyABCD*, *iss*, *iucABCD*, *iutA*

^a^Relaxase and replication proteins were identified by the use of homemade databases^51^. ^b^Incompatibility groups were determined according to the PBRT scheme^8^ and pMLST subtypes according to the allele scheme in http://pubmlst.org/plasmid/. ^c^Antimicrobial resistance genes were detected using the ARG-ANNOT^48^ and ResFinder^49^ databases. ^d^Virulence genes were detected according to VirulenceFinder^50^ and homemade databases. ^e^na, not applicable; nd, not detected. ^f^Length of the ICE (integrative and conjugative element)-containing contig. *Closed plasmids.

Overall, the seven *E*. *coli* genomes included 23 plasmids, which were classified as MOB_P51_/ColE1-like, MOB_P131_/IncL, MOB_F12_/IncF, MOB_F11_/IncN, MOB_P3_/IncX and MOB_P12_/IncI (Table 2). For one MOB_Q12_ plasmid the incompatibility group was not determined and two other plasmids could not be affiliated with any of the MOB categories. Finally, a MOB_H2_ relaxase was identified in the chromosome of *Ec*-HUCA 1, consistent with the presence of an integrative and conjugative element.

**Figure 1:**
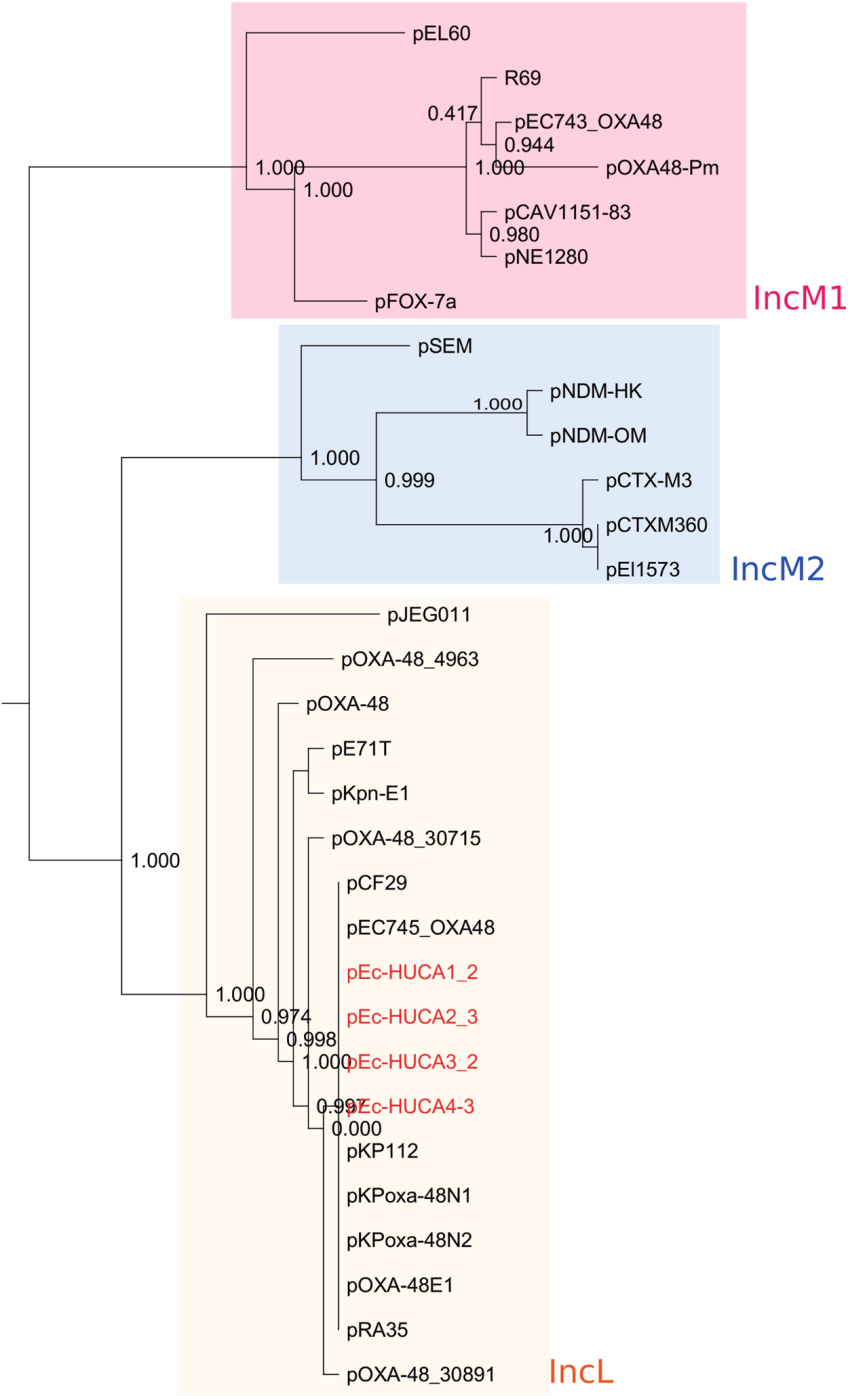
Phylogenetic tree of IncL plasmids from OXA-48-producing isolates of *Escherichia coli*. The tree is based on SNPs found in the core genome (19,995 bp +/− 45 bp; 26 CDS with ≥80% identity, ≥60% pairwise alignment coverage), common to 31 IncL (18), IncM1 (seven) and IncM2 (six) plasmids. Bootstrap support values of 1,000 replicates are shown at the nodes. Clusters corresponding to each group/subgroup are enclosed in yellow, pink and blue boxes. IncL plasmids are more closely related to IncM2 than to IncM1 plasmids.

**Figure 2:**
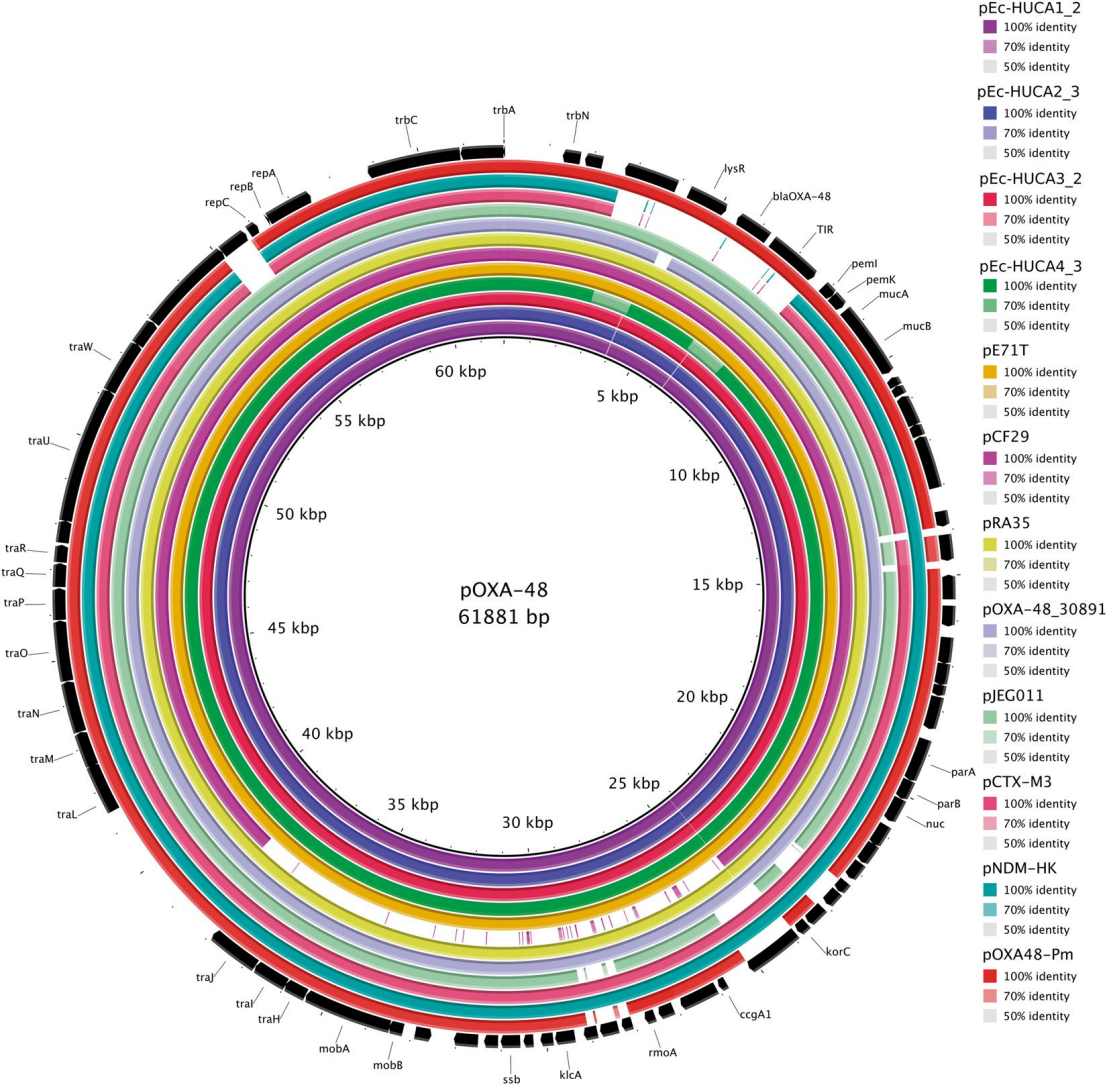
BRIG (Blast Ring Image generator) comparison of the IncL plasmids from OXA-48-producing *Escherichia coli* isolates and representative IncL, IncM1 and IncM2 plasmids. Each ring corresponds to a plasmid (indicated at the right of the figure together with the color code). Plasmid pOXA-48 (inner black ring) was used as a reference. Their genes are shown in the outer ring, represented by black arrows that also indicate the direction of transcription.

In four out of the seven *E*. *coli* isolates (*Ec*-HUCA 1 to 4; Table 2), the MOB_P131_/IncL plasmids were experimentally and *in silico* linked with the *bla*_OXA-48_ gene. Their genetic relationship was assessed by building a phylogenetic tree, which also included other IncL and IncM plasmids present in the GenBank-NCBI database (Fig. 1). Assignment to one or the other Inc group was corroborated *in silico*, or newly determined using the primers reported by Carattoli *et al*.^8^. The tree separated IncL and IncM plasmids in two clusters, the latter with two subclusters, IncM1 and IncM2, as previously observed^8^. Interestingly, *bla*_OXA-48_ was not only carried by IncL plasmids but also by IncM1 plasmids, formerly classified as IncL/M^24,25^. Besides, it was found also on IncA/C plasmids^26^. The backbones of the IncL plasmids from the HUCA isolates were identical to each other and to nine other IncL plasmids, originating in *Klebsiella pneumoniae*, *Citrobacter freundi*, *Raoultella planticola* and *E*. *coli*. These results underline the prevalence of an IncL plasmid lineage which plays a major role in the horizontal spread of *bla*_OXA-48_ between members of the *Enterobacteriaceae* ^5^. BRIG comparison of the IncL plasmids is shown in Fig. 2. The p*Ec*-HUCA and pE71T^27^ plasmids are highly similar to pOXA-48^11^. pCF29 (Accession Number LN864820) showed a large gap at the mobilization-transfer region (24–39 Kb); whereas pCTX-M3^28^ and pNDM-HK^29^ lack the *bla*_OXA-48_ region (2.5–8 Kb), as expected.

### Genetic environment of the bla_OXA-48_ gene

**Figure 3:**
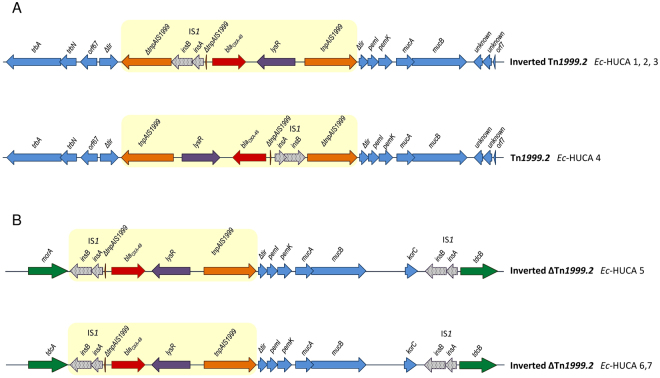
Genetic environment of the *bla*_OXA-48_ gene. (**A**) Structures of the Tn*1999*-like transposons and adjacent DNA from the IncL *bla*_OXA-48_ plasmids of *Ec*-HUCA 1, 2, 3 and 4. (**B**) Structure of the inverted and deleted Tn*1999*.*2* transposon and the adjacent DNA including the insertion sites in the chromosomes of *Ec*-HUCA 5, 6 and 7. Open reading frames are represented by arrows indicating the direction of transcription and having different fillings: orange, IS*1999*; black dots, IS*1*; red, *bla*_OXA-48_; purple, *lysR*; blue, IncL plasmid genes; green, *E*. *coli* chromosomal genes. The Tn*1999*-like structures are highlighted by yellow boxes.

As already indicated, *bla*_OXA-48_ was carried by MOB_P131_/IncL plasmids in four *E*. *coli* isolates, but chromosomally located in another three (Table 2). Although the DNA surrounding *bla*_OXA-48_ could not be assembled from the short Illumina reads, the location of the gene within Tn*1999*-like transposons and the genetic environment of the transposons was established by PCR mapping. Specifically, *bla*_OXA-48_ was carried by Tn*1999*.*2* in *Ec*-HUCA 4; an inverted Tn*1999*.2 in *Ec*-HUCA 1 to 3; and an inverted and deleted version of Tn*1999*.2 in *Ec*-HUCA 5 to 7 (Fig. 3). Tn*1999*.2 differs from Tn*1999*.*1* by insertion of IS*1R* into the IS*1999* copy upstream of *bla*_OXA-48_, while in the deleted version, the IS*1R*-containing copy of IS*1999* is truncated at the 5′ end^9^. IS*1R* enhances transcription of the downstream *bla*_OXA-48_ gene by providing an efficient promoter −35 box with an optimal 17-bp spacing with regard to the −10 box supplied by IS*1999* ^12^. This fact could explain the more frequent detection of Tn*1999*.2 and variants in comparison with Tn*1999*.*1* ^6^. Recently, the complete sequences of the first two ST131 *bla*_OXA-48_ plasmids have been reported^25^; in both, the gene was carried by Tn*1999*.2, like in *Ec*-HUCA 4.

To identify the insertion sites of *bla*_OXA-48_ within the chromosomes of *Ec*-HUCA 5 to 7, assembled contigs were analysed. Three contigs containing sequences homologous to pOXA-48a (NC_019154^11^) and pRA35 (LN864821^9^) were identified, and two of them were flanked by *E*. *coli* chromosomal genes. The three isolates shared an identical 21.9 Kb fragment in which the inverted and deleted version of Tn*1999*.2 was found, followed by genes previously detected in pOXA-48a and pRA35. This fragment, which is flanked by IS*1R*, constitutes an IS*1R* composite transposon, termed Tn*6237* ^9^. With the single exception of an isolate from Lebanon^9^, this is the first report of a Tn*6237* insertion into the chromosome of ST131. The precise insertion site of the transposon was established by PCR mapping (Supplementary Figure S4). In *Ec*-HUCA 6 and 7, Tn*6237* was placed between the *tdcA* (DNA-binding transcriptional activator) and *tdcB* (threonine dehydratase) genes, which are contiguous in other *E*. *coli* genomes. In the case of *Ec*-HUCA 5, Tn*6237* was followed by *tdcB* but *morA* (putative dehydrogenase) was placed upstream. This organization may have resulted from inversion of the chromosomal segment spanning *tdcA* to *morA*. Consistent with its expected lack of specificity, IS*1*-flanked Tn*6237*, was also found in three other insertion sites within the *E*. *coli* chromosome, in isolates from the United Kingdom, Czech Republic and Lebanon^9,30,31^.

### Resistance genes for other β-lactam and non-β-lactam antimicrobials

According to their phenotypes (Table 1), additional resistance genes were found in all OXA-48 isolates, contained in MOB_F12_/IncF, MOB_F12_/IncF + IncI1 and MOB_F11_/IncN plasmids or in the chromosome (Table 2). Thus, diverse plasmids act as vehicles for the spread of resistance genes between *E*. *coli* clones/subclones circulating in the same health area. Remarkably, *bla*_CTX-M-15_ appeared in different locations within the four ST131 isolates carrying it. Specifically, *bla*_CTX-M-15_ was chromosomally located in two out of the three isolates belonging to the *H*30-Rx subclone (*Ec*-HUCA 5 and 6), whereas in *Ec*-HUCA 7 it was carried by a MOB_F11_/IncN (ST7) plasmid of 48.7 Kb, together with *qnrS1* and *dfrA14*, for plasmid-mediated quinolone resistance (PMQR) and trimethoprim resistance, respectively. Interestingly, a slightly smaller MOB_F11_/IncN (ST7) plasmid of 45.8 Kb contained *qnrS1* and *dfrA14*, but not *bla*_CTX-M-15_, in *Ec*-HUCA 6. The *bla*_CTX-M-15_ gene of the non-ST131 isolate (*Ec*-HUCA 2), was carried by a conjugative MOB_F12_/IncF plasmid of ca. 150 Kb^15^.

In the three ciprofloxacin resistant ST131 *H*30*-*Rx-OXA-48 isolates (*Ec*-HUCA 5 to 7; MIC > 32 mg/L), *in silico* analysis of the genomes revealed the distinct *gyrA*/*parC* allele combination (*gyrA*-S83L/D87N; *parC*-S80I/E84V) previously reported for the *H*30 subclone^32^. The S83L mutation was also found in the *gyrA* gene of *Ec*-HUCA 1 and 4, which were resistant to nalidixic acid but susceptible to ciprofloxacin. Apart from *qnrS1* found in *Ec*-HUCA 6 and 7, other PMQR genes, including additional *qnr* genes and *qep* or *oqx* genes, tested negative by bioinformatic methods. The genetic bases of most other resistances could be also established by *in silico* analysis (Tables 1 and 2), being of note the presence of a W30R mutation in the *folA* gene (dihydrofolate reductase) of *Ec*-HUCA 5, which was resistant to trimethoprim-sulfamethoxazole but lacked *sul* and *dfr* genes.

### Virulence gene content and studies of virulence “*in vivo*”

Based on PCR-screening of 50 genes/alleles characteristic of ExPEC, *Ec-*HUCA 2 and 4 showed the lowest and highest virulence scores, with 16 and 33 virulence factors (VF), respectively. Of the five ST131 isolates, *Ec*-HUCA 4 was virotype D5 while *Ec*-HUCA 5 to 7 were virotype E (Supplementary Figure S1; Supplementary Table S1). The virulence profile of the O16:H5-ST131 isolate did not match any of the 12 virotypes included in the scheme^33^. Four out of the five ST131 (*Ec-*HUCA 4 to 7) and the ST83 (*Ec-*HUCA 3) isolates showed the ExPEC status, and all isolates except *Ec-*HUCA 1 were classified as UPEC.

PCR-screening results were complemented by *in silico* analysis of the genome sequences, which allowed the identification of additional VFs, mainly encoded by genes located in the chromosome but also on plasmids (Table 2). Like before, the highest VF score was obtained for *Ec*-HUCA 4, followed by the ST131 *H*30-Rx-OXA-48 isolates (*Ec*-HUCA 5 to 7), which shared the same profile consisting of four operons and 15 individual genes. In contrast, a score of 16 was obtained for *Ec*-HUCA 1, the ST131 isolate with undefined virotype.

With regard to non-ST131 isolates, 27 VFs were detected in *Ec*-HUCA 2 and only 13 in *Ec*-HUCA 3. Interestingly, the plasmid virulence genes of *Ec*-HUCA 2 and 4 were nearly identical, and some virulence genes have chromosomal and plasmid copies (*iss* in *Ec*-HUCA 2, and *mchF* and *iss* in *Ec*-HUCA 4), providing regions of homology for interaction between the two replicons.

Finally, the intrinsic extraintestinal virulence of the OXA-48-producing *E*. *coli* was assessed in a mouse sepsis model. Within a seven day experiment, all mice challenged with *E*. *coli* CFT703 (positive control) died, compared with none of the mice challenged with *E*. *coli* MG1655 (negative control), which remained healthy. All OXA-48 isolates showed lethality as low as ≤10% and only three (*Ec*-HUCA 2, 6 and 7) caused the death of one out of the ten inoculated mice. In contrast, all isolates caused local inflammatory response, with extensive subcutaneous necrosis, in the surviving mice (Supplementary Table S2). Previously, we observed different virulence patterns in the final lethality, the rapidity in causing death and the inflammation-causing ability of ST131 isolates in correlation with the virotype, with the highest lethality (≥80% of mice challenged killed) shown by virotypes A, B, C and D1. By contrast, isolates within virotypes D2, D3 and D4 led to different outcomes, and isolates of virotype E showed the lowest final lethality, varying from 10 to 40% of the challenged mice. We also observed that certain ST131 isolates of virotypes C, D, and E induced an acute inflammatory response in the inoculation region^22^, like those in this study. There are few comparable *in vivo* studies and this is the first one assaying OXA-48-producing isolates. Results derived from the sepsis model might be consistent with the clinical nature of the isolates, not involved in severe disease but recovered from surgical wounds and UTIs. Future studies would be necessary to investigate the mechanisms responsible for the differences in lethality within ST131 virotypes.

### Phylogenomics of the OXA-48-producing isolates

**Figure 4:**
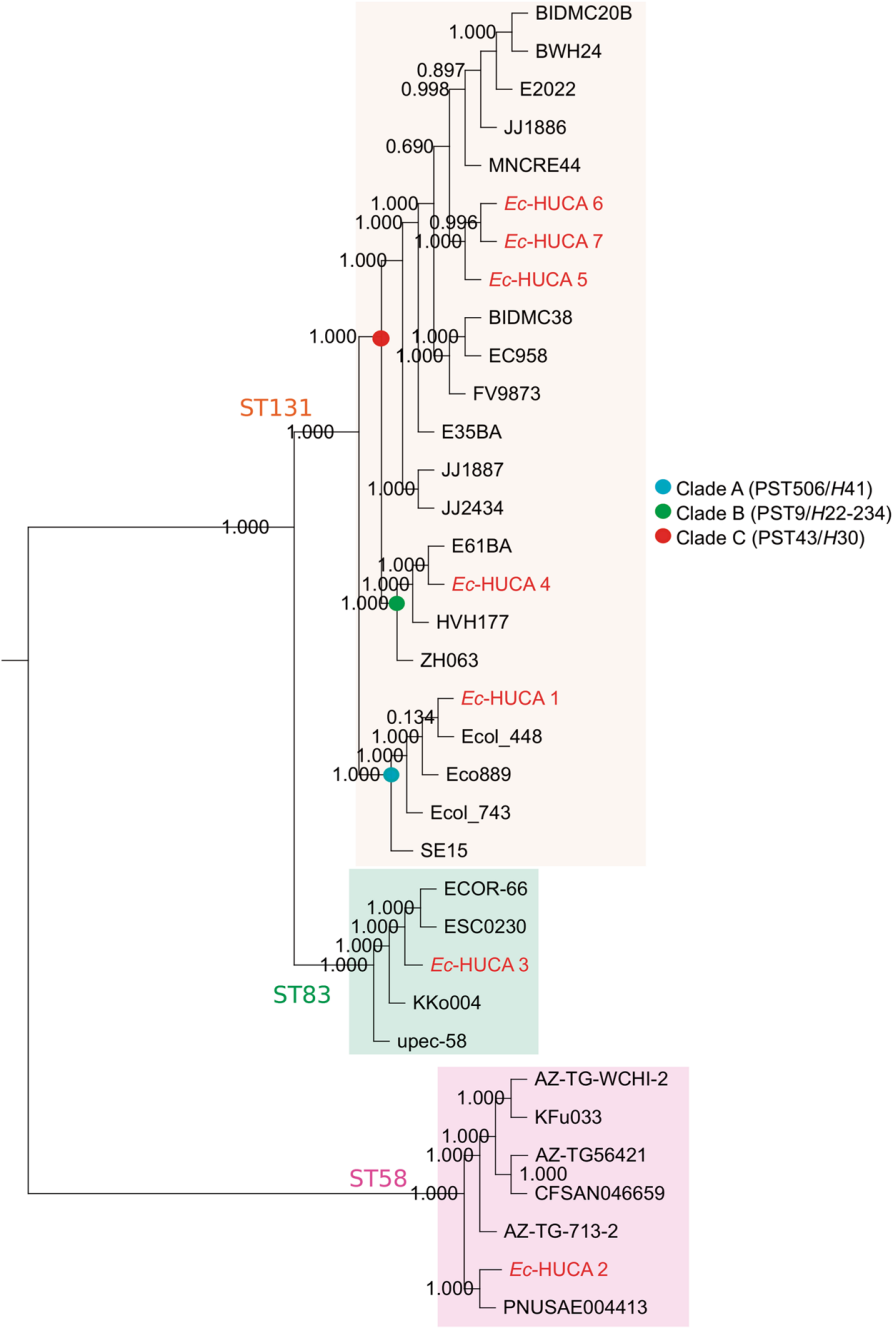
Phylogenetic tree of the OXA-48-producing *Escherichia coli* genomes. The tree is based on the core genome SNPs (3,154,218 bp ± 1716 bp; 3185 CDS with ≥80% identity, ≥60% pairwise alignment coverage). Bootstrap support values of 1,000 replicates are shown at the nodes. The ST131 clades are indicated with blue (Clade A, PST506/*fimH*41), green (Clade B, PST9/*fimH*22-234) and red (Clade C, PST43/*fimH*30) circles.

The phylogenetic context of the OXA-48-producing *E*. *coli* was assessed by comparison with 28 *E*. *coli* genomes with different STs, PSTs, virotypes and *fimH* alleles (Fig. 4, Supplementary Table S3). All ST131 isolates grouped in a single cluster which was further divided into subclusters according to clade, virotype and serotype. In agreement with their assignment to phylogroup B2, the ST131 cluster was genetically closer to ST83 (also B2) than to ST58 (phylogroup B1). It is of note that only two SNPs differences were found between the COG-based core genomes of *Ec*-HUCA 6 and 7, and that both differed from *Ec*-HUCA 5 by 14 SNPs. Comparing these three highly similar isolates using SNP analysis and conventional PFGE, we found that both approaches discriminated between *Ec*-HUCA 5 *vs* 6 and 7, the latter displaying 100% identity between them and 88.4% with *Ec*-HUCA 5 by PFGE (Supplementary Figure S1). As shown in Table 1, these clonal isolates were detected in a primary-care centre, in the emergency unit of the HUCA and in the geriatric unit of a long-term facility. Thus, the *H*30-Rx-OXA-48 subclone, with the chromosomally located carbapenemase gene, is circulating both in the community and health-care institutions, which indicates transmission between the two settings. The number of SNPs between these and other isolates progressively increased according to the ST/PST and phylogroup affiliation, with averages of 3,755, 8334, 19,787 and 79,640, with regard to *Ec*-HUCA 4, 1, 3 and 2, respectively.

Concerning *Ec-*HUCA 5, 6 and 7, they are classified as virotype E and belong to clade C (PST43/*H*30), which comprises previously reported ST131 genomes with virotypes A, B and C^23^. None of these ST131 references carried the *bla*_OXA-48_ gene, although BIDMC20B, BWH24 and MNCRE-44 (virotype C) harbour the *bla*_KPC-3_ carbapenemase gene, always located on large conjugative plasmids^23,34,35^. It is important to note that this is the first study where ST131 virotype E genomes have been sequenced. In the clades A (PST506/*H*41) and B (PST9/*H*22-234), containing the *Ec*-HUCA 1 and 4 genomes, respectively, only Ecol_743 harbours the *bla*_OXA-48_ gene (located on a 69 Kb plasmid named pEC743_OXA48), while Ecol_448 contains the closely related class D beta-lactamase *bla*_OXA-163_ gene (pEC448_OXA163, 71 Kb plasmid)^25^.

According to our results, diverse OXA-48-producing *E*. *coli* clones are circulating in Oviedo, a situation justified in part by the conjugative potential of the IncL plasmid carrying the *bla*_OXA-48_ gene. Additional plasmids also play a role as vehicles of resistance and/or virulence genes. Treatment of the affected patients represents a serious challenge since all except one isolate were MDR. So, antimicrobial stewardship policies, new antimicrobial therapy approaches and control measures are necessary to combat the infections caused by these bacteria, and to control further dispersal.

## Material and Methods

### Epidemiological background of the OXA-48-producing isolates

The isolates were recovered from wound infections or UTIs between 2012 and 2015 (Table 1). Like *Ec*-HUCA 1 to 3^15^, *Ec*-HUCA 4 caused a hospital-acquired infection and affected a critical patient exposed to long-term hospitalization and prolonged antimicrobial treatment in the HUCA. The remaining isolates were recovered from patients attended at a primary care centre (*Ec*-HUCA 5), the emergency unit of the HUCA (*Ec*-HUCA 6), and the geriatric unit of a long-term care facility associated with the hospital (*Ec*-HUCA 7).

### Antimicrobial susceptibility testing and plasmid analysis

For the new isolates, antimicrobial susceptibility was determined by disk (Oxoid, Madrid, Spain or Becton Dickinson, Sparks, MD, USA) diffusion assays and the Microscan system (MicroScan, Beckman Coulter, CA, USA), which also allows bacterial identification. MICs for erythromycin were determined by broth microdilution following CLSI guidelines^36^. MICs for carbapenems (ertapenem, imipenem and meropenem) were obtained with Etest strips (bioMérieux, Marcy-l’Étoile, France). Results were interpreted according to CLSI breakpoints^36^. Carbapenemase production was confirmed by the modified Hodge and Carba NP tests^37^. Identification of genes encoding resistance to carbapenems and broad-spectrum cephalosporins, and plasmid analysis were performed as reported^8,15,38^. The genetic context of the *bla*_OXA-48_ gene was determined by PCR mapping (see Supplementary Table S4 and Supplementary Figure S4).

### Typing, subtyping and phylogenetic grouping of the isolates

*E*. *coli* isolates were characterized with regard to O:H serotype and *fimH* alleles (for type 1 fimbrial adhesion)^39–41^. The STs were established following the MLST schemes of Achtman (http://mlst.warwick.ac.uk/mlst/dbs/Ecoli) and the Pasteur Institute (http://bigsdb.pasteur.fr/ecoli/ecoli.html). The *Xba*I-PFGE profiles were determined according to PulseNet protocol (http://www.pulsenetinternational.org/), imported into BioNumerics (Applied Maths, St-Martens-Latern Belgium) and clustered by Dice/UPGMA. Fifty genes/alleles encoding virulence factors (VF) were screened by PCR (Supplementary Table S5). Isolates were presumptively designated as ExPEC if positive for two or more of five markers and as uropathogenic (UPEC) if positive for three or more of four markers (Supplementary Table S1)^42,43^. The virotype of the ST131 isolates was established according to the scheme described by Dahbi *et al*.^33^. Assignment to the main phylogroups (A, B1, B2 and D) was based on the protocol of Clermont *et al*.^17^.

### Genome sequencing, assembly and analysis

Total DNA from *E*. *coli* isolates was extracted with the QIAmp DNA Mini Kit (Qiagen). Libraries were prepared using the TruSeq PCR-free DNA Sample Preparation Kit (Illumina) at the sequencing facility of the University of Cantabria. Paired-end 100 bp reads (550 bp insert size) were sequenced in a HiSeq. 2500 (Health in Code Facility). Reads were assembled with the VelvetOptimiser.pl script of Velvet software^44^. Serotype, MLST, *fimH* alleles and virulence gene profiles were *in silico* determined with SerotypeFinder v1.1^45,46^, and homemade MLST (Achtman and Pasteur schemes), *fimH* and virotype databases^47^. Antimicrobial resistance genes were detected using the ARG-ANNOT^48^ and ResFinder^49^ databases; virulence gene content was established with VirulenceFinder and homemade databases^50^.

For phylogenetic analysis, core genome was defined as described by Lanza *et al*.^23^, using the genomes of the *E*. *coli* isolates from the HUCA plus reference full-genomes retrieved from GenBank-NCBI (ftp://ftp.ncbi.nlm.nih.gov/genomes/genbank/bacteria/) and Enterobase (http://enterobase.warwick.ac.uk/species/index/ecoli).

### Plasmid reconstruction from WGS data and analysis

Plasmid reconstructions were based on the PLACNET method^23^. Contig analysis was performed against complete bacterial genomes and plasmids from GenBank-NCBI. Relaxase proteins (REL) and Replication Initiation Proteins (RIP) were identified using in-house databases^23,51^. Incompatibility groups and pMLST subtypes were experimentally^8,52^ and/or *in silico* determined (http://pubmlst.org/plasmid/). Reconstructed *bla*_OXA-48_ IncL plasmids and references belonging to the IncL and IncM groups were compared using BRIG^53^, and a phylogenetic tree was built from variable positions (SNPs) in genes encoding core proteins.

### Mouse lethality assay

A mouse sepsis model was used to assess extraintestinal virulence of the isolates^22^. For each one, 10 outbred female RjOrl:Swiss mice (3–4 weeks old; Janvier Labs, France) received a subcutaneous injection into the nape of the neck of approximately 2 × 10^8^ CFU of log-phase bacteria. After inoculation, mice were clinically inspected along one week. Time of death and local presence of lesions (acute inflammation in the region of inoculation) were recorded for each mouse. Surviving mice were euthanatized on day seven by cervical dislocation. In each assay, two control isolates were included: *E*. *coli* K-12 MG1655 which does not kill mice by seven days post-challenge, and *E*. *coli* CFT073 which shows a lethality of ≥80% by seven days post-challenge. Results of lethality were indicated as the number of mice killed within 24 h and within seven days post-injection.

### Ethics statement

All experimental protocols dealing with bacteria from human samples were approved by the ethics committee of the HUCA.

All animal experimentation was conducted following European (Directive 2010/63/EU on the protection of animals used for scientific purposes) and National (RD 53/2013) regulations for transport, housing and care of laboratory animals. The protocol used was approved by the Animal Welfare Committee of the Veterinary Faculty in Lugo, University of Santiago de Compostela (AE-LU-002/14-1).

### Data availability

This Whole Genome Shotgun project has been deposited at DDBJ/ENA/GenBank under accession numbers NBSZ00000000, NBSY00000000, NBSX00000000, NBSW00000000, NBSV00000000, NBSU00000000, NBST00000000 for *Ec*-HUCA 1 to 7, respectively. All the samples are part of BioProject PRJNA381431 and correspond to BioSample IDs SAMN06676454 to SAMN06676460.

## Electronic supplementary material

Supplementary Information
