# Supplementary material for: Whole genome sequencing, molecular typing and in vivo virulence of OXA-48-producing Escherichia coli isolates including ST131 H30-Rx, H22 and H41 subclones
Source: Sci Rep. 2017 Sep 21;7:12103. doi: 10.1038/s41598-017-12015-0 (PMC5608912; doi:10.1038/s41598-017-12015-0)
Supplement: Supplementary file 1 — Supplementary Information [file 41598_2017_12015_MOESM1_ESM.pdf]

Supplementary Information for

Whole genome sequencing, molecular typing and *in vivo* virulence of OXA-48-producing *Escherichia coli* isolates including ST131 *H*30-Rx, *H*22 and *H*41 subclones

María de Toro[1,#], Javier Fernández[2,3#], Vanesa García[2,4], Azucena Mora[4], Jorge Blanco[4], Fernando de la Cruz[5] and M. Rosario Rodicio[2,*]

[1] Plataforma de Genómica y Bioinformática, Centro de Investigación Biomédica de La Rioja (CIBIR), Logroño, Spain; [2]Departamento de Biología Funcional, Área de Microbiología, Universidad de Oviedo (UO), Oviedo, Spain; [3]Servicio de Microbiología, Hospital Universitario Central de Asturias (HUCA), Oviedo, Spain; [4]Laboratorio de Referencia de Escherichia coli (LREC), Departamento de Microbioloxía e Parasitoloxía, Facultade de Veterinaria, Universidade de Santiago de Compostela (USC), Lugo, Spain, [5]Departamento de Biología Molecular and Instituto de Biomedicina y Biotecnología de Cantabria (IBBTEC), Universidad de Cantabria-CSIC, Santander, Spain

[#]Both authors have equally contributed to this work.

Corresponding author. M. Rosario Rodicio. Departamento de Biología Funcional, Área de Microbiología, Universidad de Oviedo, 33006 Oviedo, Spain. E-mail: rrodicio@uniovi.es

Supplementary Figures

Supplementary Figure S1. Pulsed-field gel electrophoresis of *Xba*I-digested DNA from the seven OXA-48 *Escherichia coli* isolates. The dendrogram was obtained with the UPGMA algorithm based on the Dice similarity coefficient and applying 1% of tolerance in the band position. On the right of the dendrogram, isolate designation, O:H serotype, virotype, ST, PCR typing of *bla* genes and filogroup. The virotype of the ST131 isolates was established based on the presence or absence of 13 VF according to the scheme described by Dahbi *et al.*[1] with 12 defined virotypes. Of the five ST131 isolates of the study, *Ec*-HUCA 4 showed virotype D5 (positive for VF *papG III*, *cnf1*, *hlyA*, *iroN*, *kpsM II-K5* and *ibeA*) and *Ec*-HUCA 5 to 7 showed virotype E (positive for VF *papG II*, *cnf1*, *sat*, *hlyA*, and *kpsM II-K5*). Virulence profile of the O16:H5-ST131 isolate did not match any virotype of the scheme, so it was showed as NT (not typeable); NA (not applicable) for those non-ST131 isolates.

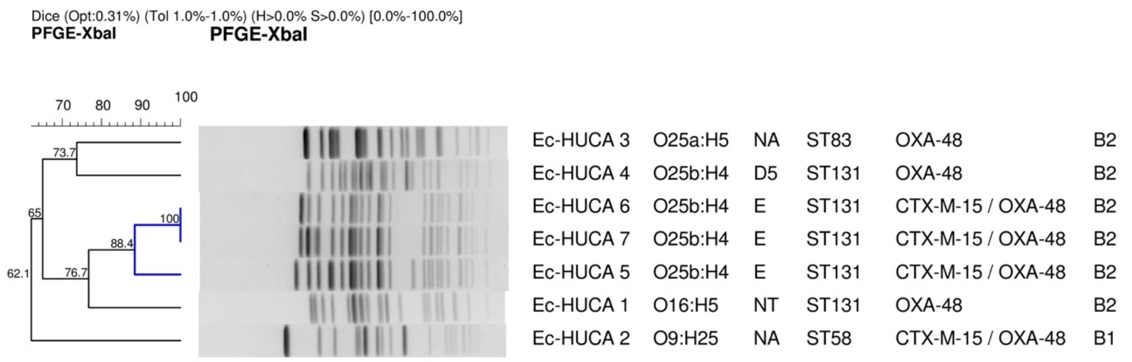

Supplementary Figure S2. PLACNET plasmid reconstructions for the seven *Ec*-HUCA genomes (*a* - *g*). Networks contain nodes of two different colors (blue for contigs, grey for reference genomes). The size of reference nodes is always the same. The size of the contig nodes is proportional to the contig length. Besides, outlines are yellow for contigs containing RIP proteins, red for relaxases and green for both proteins. Edges are either solid (scaffold links) of dotted (homologous references). The black line surrounding plasmids composed by only one contig indicates that there are closed plasmids.

Figure S2 (a)

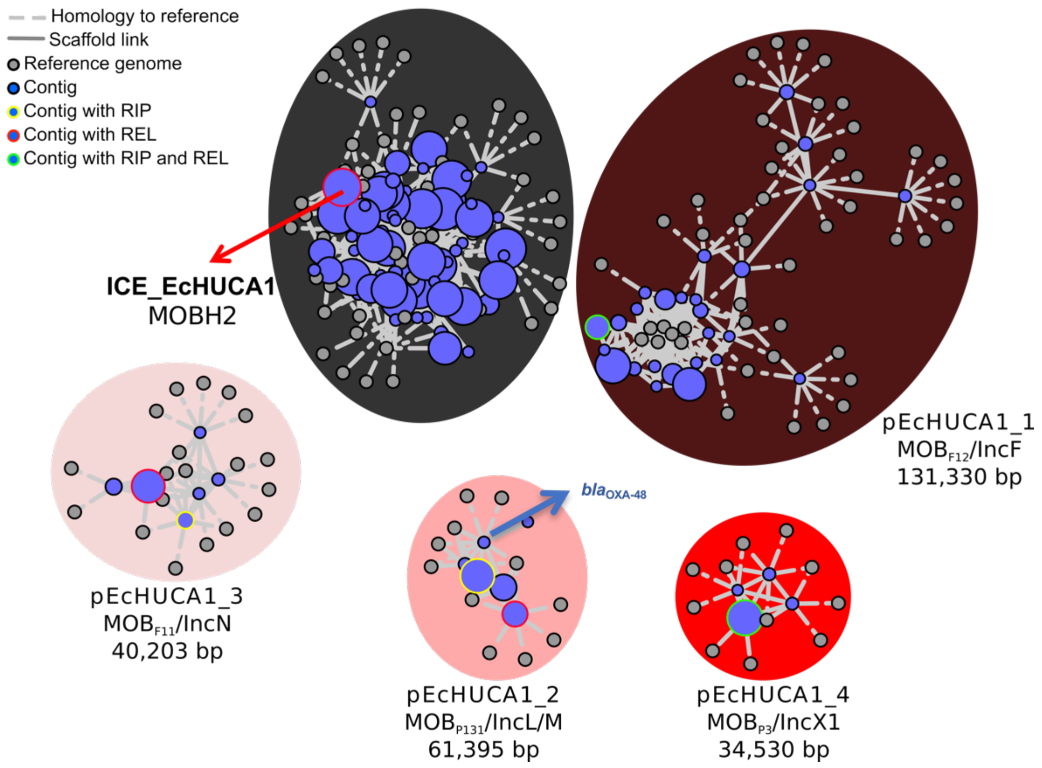

Figure S2 (b)

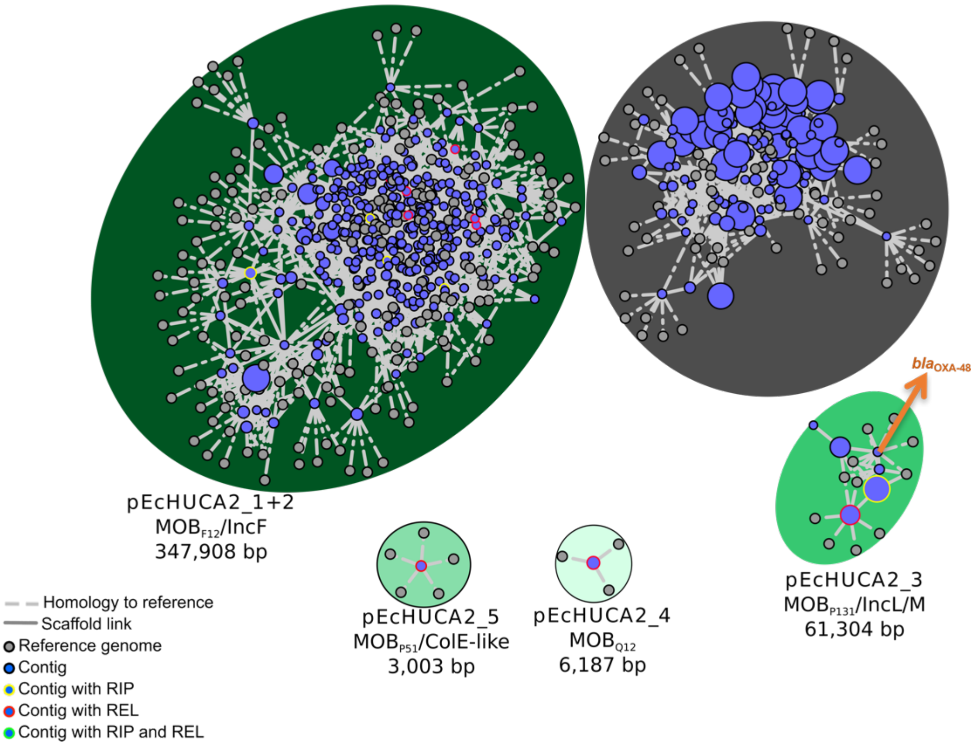

Figure S2 (c)

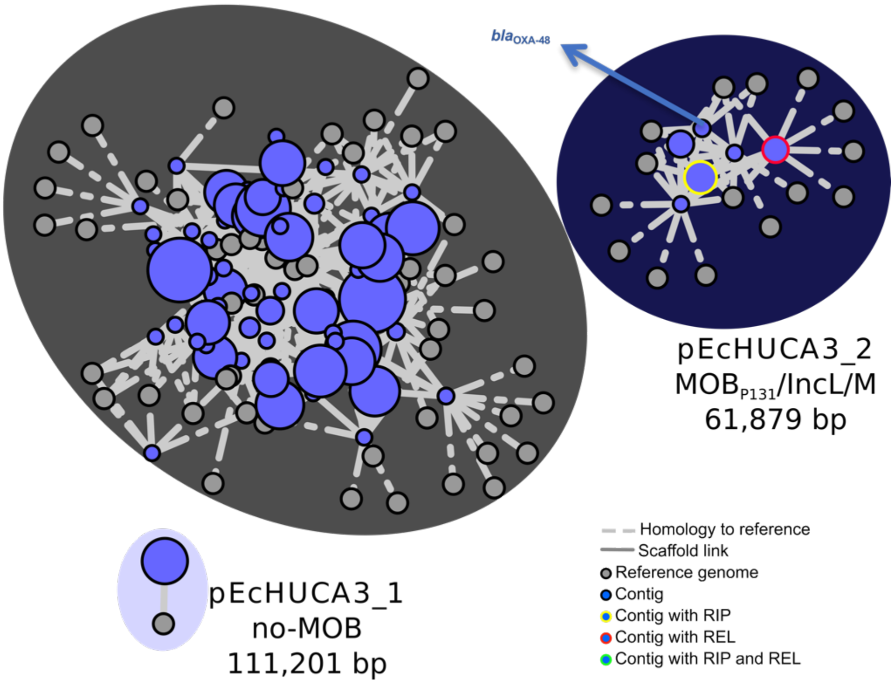

Figure S2 (d)

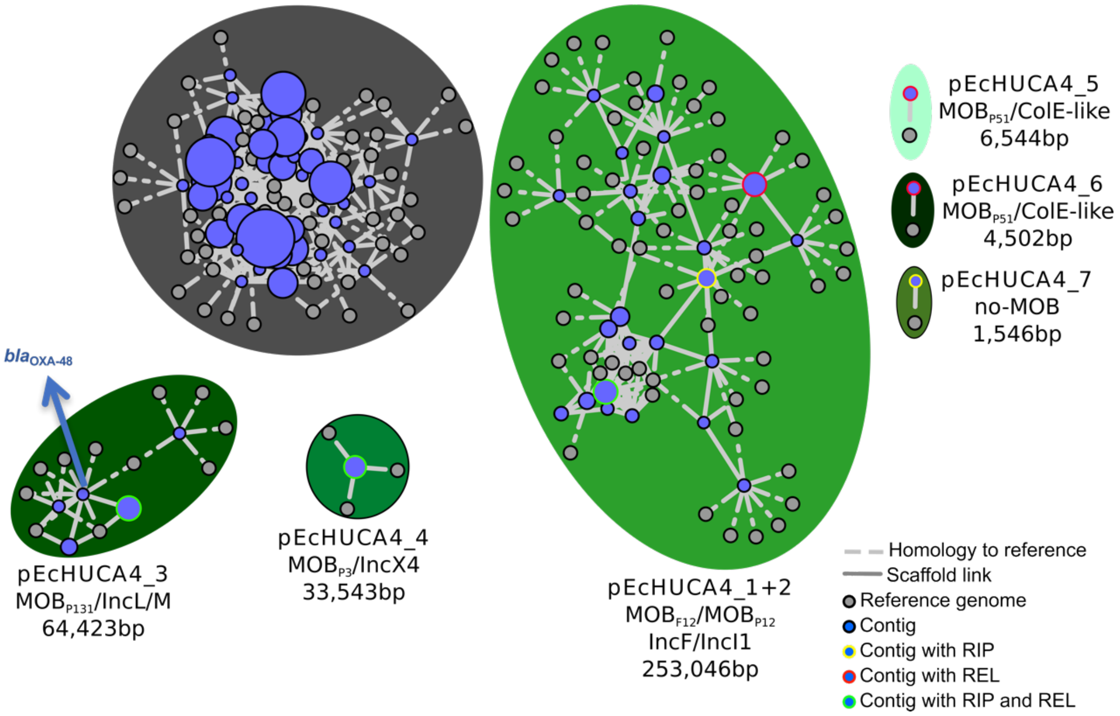

Figure S2 (e)

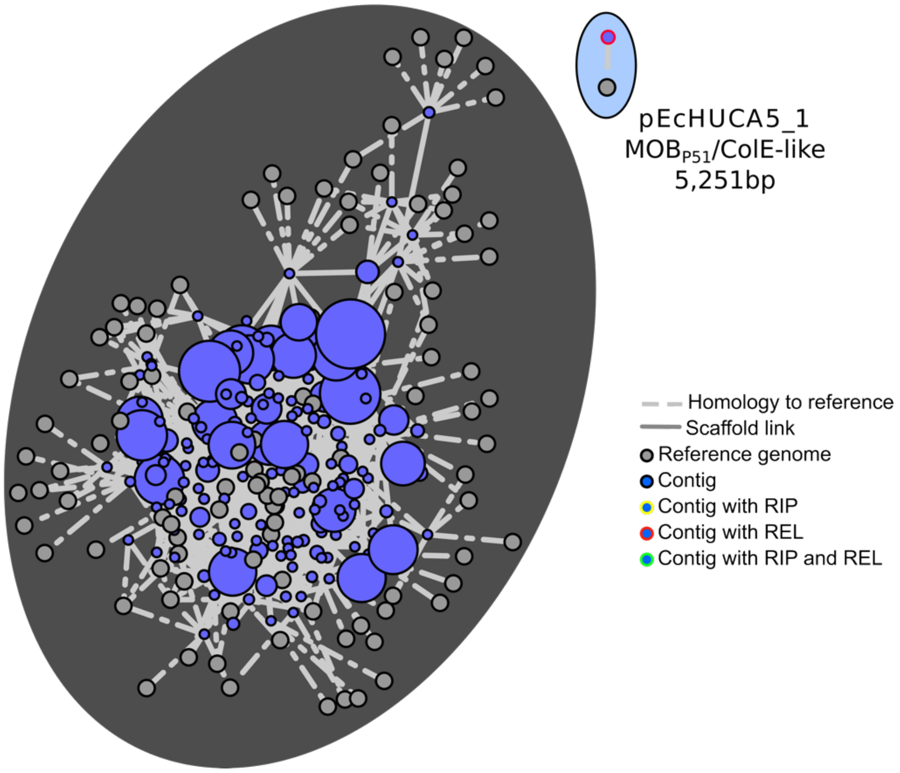

Figure S2 (f)

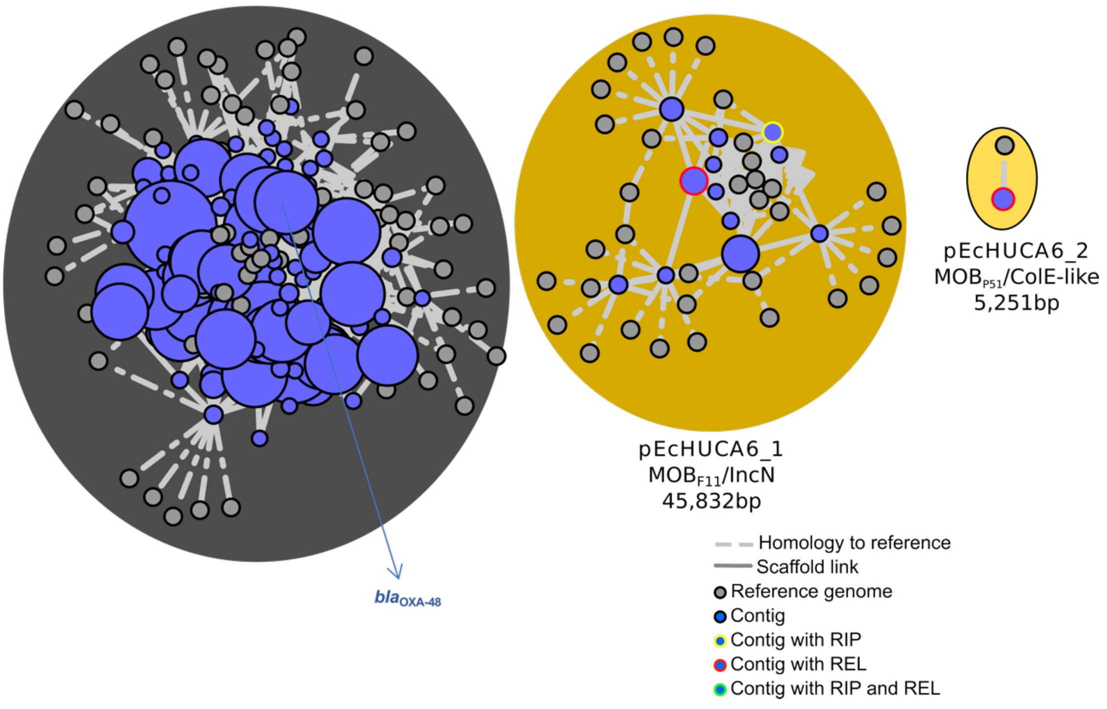

Figure S2 (g)

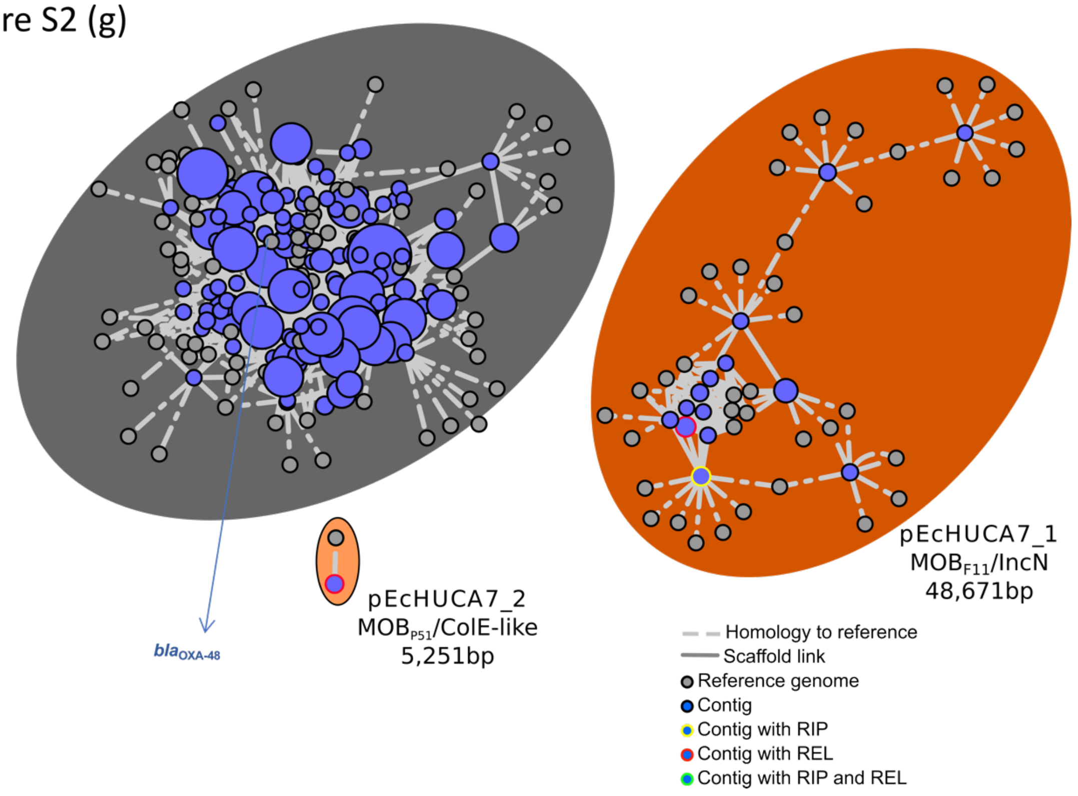

Supplementary Figure S3. Plasmid profiles of clinical isolates of *Escherichia coli* as determined by the Kado and Liu method[1]. Lane 1, *Ec*-HUCA 1; lane 2, *Ec*-HUCA 2; lane 3, *Ec*-HUCA 3; lane 4, *Ec*-HUCA 4; lane 5, *Ec*-HUCA 5; lane 6, *Ec*-HUC -6; lane 7, *Ec*-HUCA 7. Lanes i and ii, plasmids obtained from *E. coli* 39R861 (NCTC 50192) and plasmid RP4 used as molecular size standards for uncut DNA. Plasmids hybridizing with *bla*$_{OXA-48}$ probes are marked with asterisks.

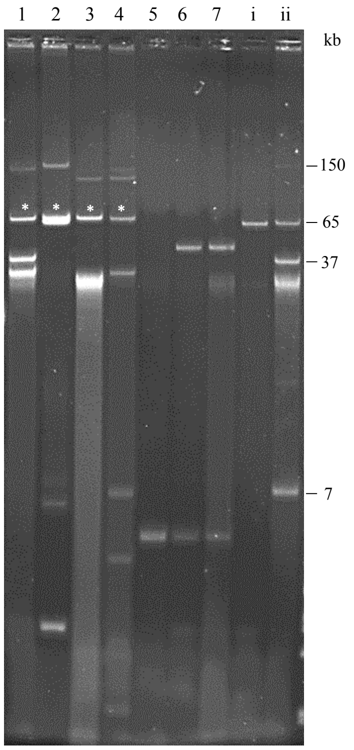

Supplementary Figure S4. Strategy used to determine the genetic environment of the *bla*$_{\text{OXA-48}}$ gene in clinical isolates of *Escherichia coli* by PCR mapping. The schematic representations of *Tn*1999.2 (A), inverted *Tn*1999.2 (B), inverted Δ*Tn*1999.2 (C and D) and *E. coli* chromosomal genes are based on GenBank accession numbers JN714122, LN864821.1 and HG977710.1, respectively. The amplicons expected in the performed reactions, and their sizes in kb, are represented by horizontal bars below the schemes, with obtained and not obtained amplicons represented by continuous and discontinuous lines, respectively. Open reading frames are represented by arrows indicating the direction of transcription and having different fillings: orange, IS*1999*; black dots, IS*1*; red, *bla*$_{\text{OXA-48}}$; purple, *lysR*; blue, IncL plasmid genes; green, *E. coli* chromosomal genes. The Tn*1999*-like structures are highlighted by yellow boxes.

A. Tn*1999.2* *Ec*-HUCA 4

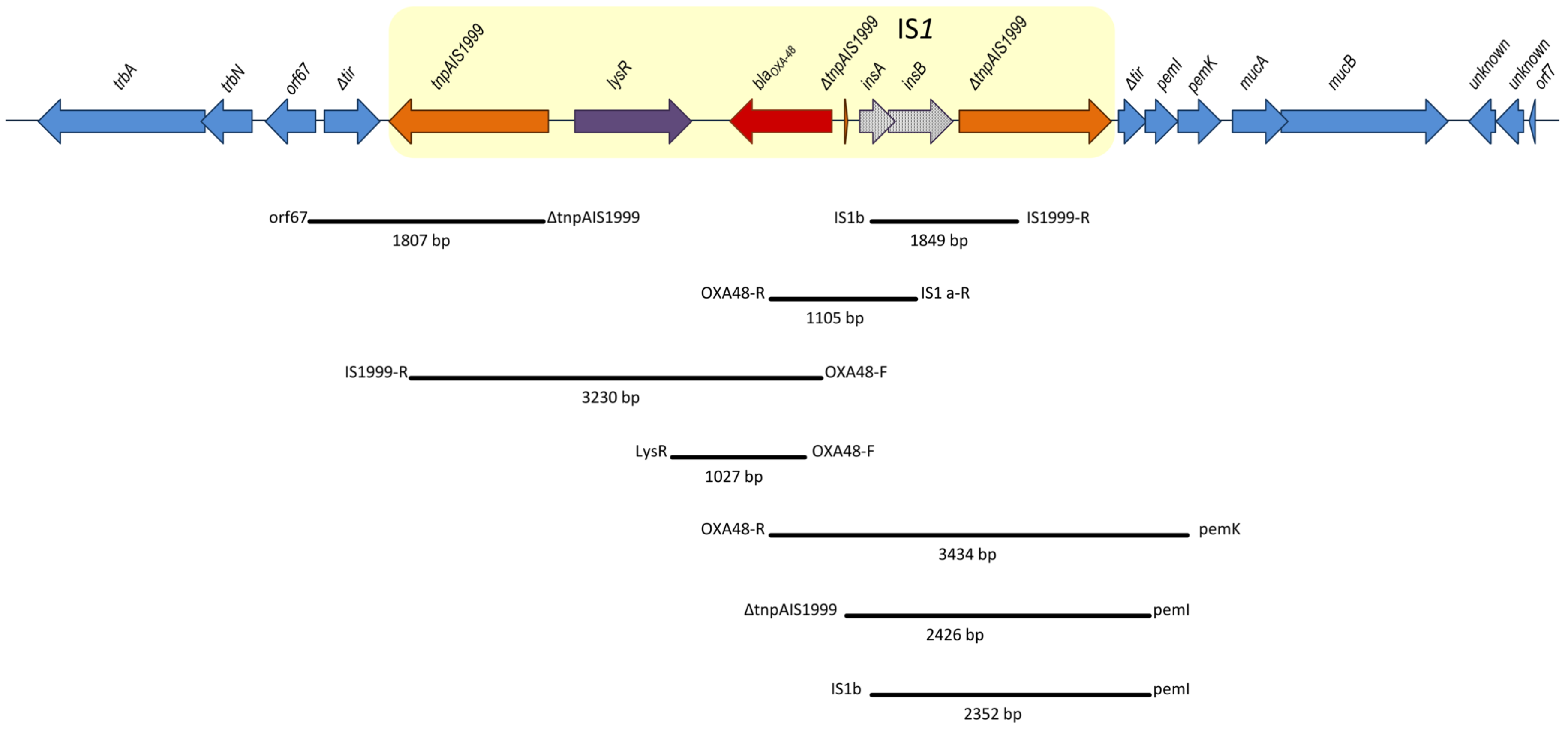

B. Inverted Tn*1999.2* *Ec*-HUCA 1, 2, 3

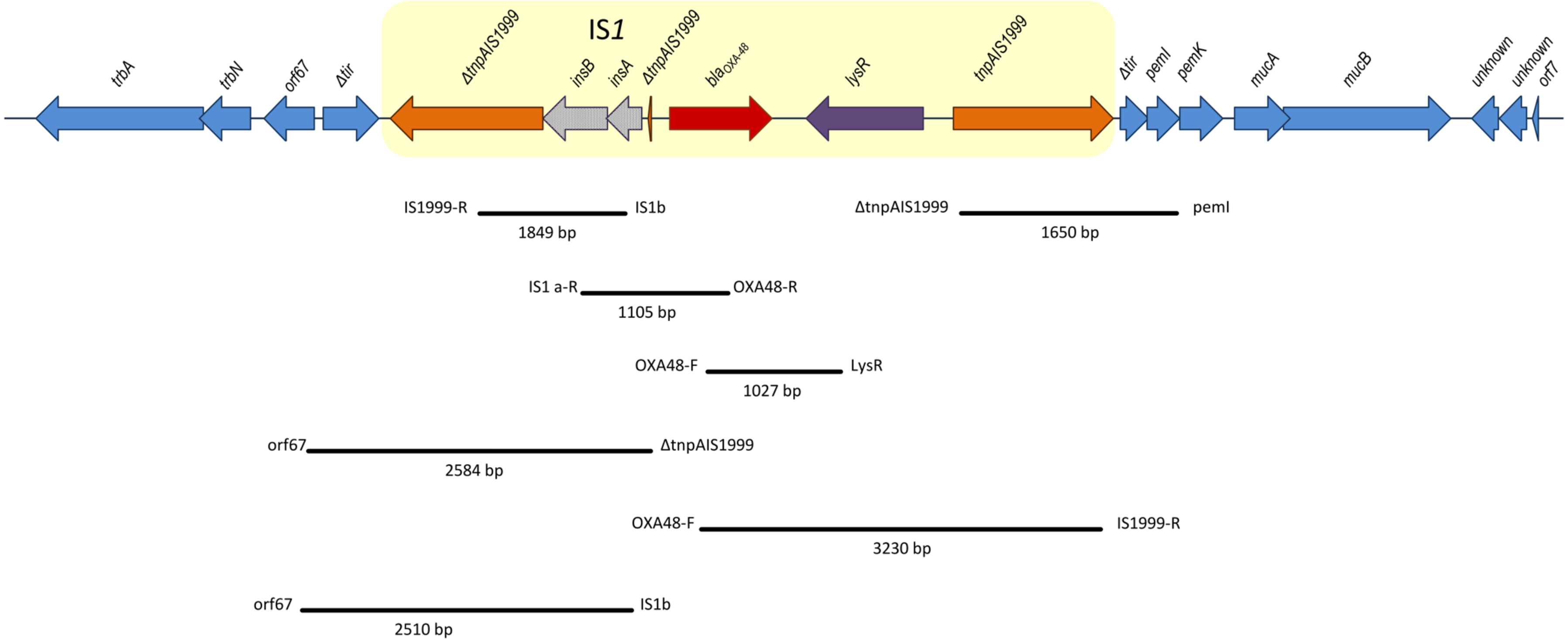

C. Inverted ΔTn*1999.2* *Ec*-HUCA 5

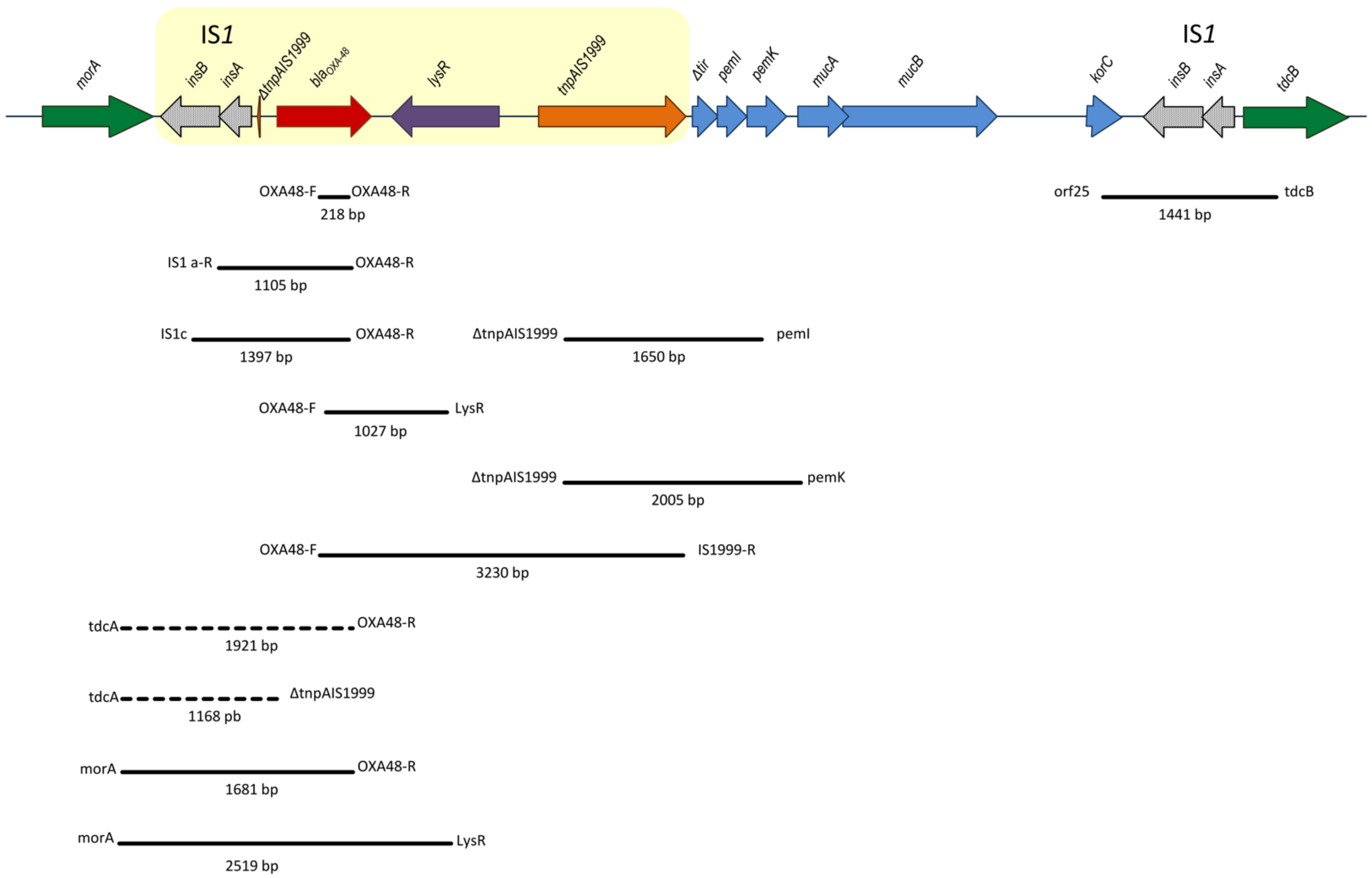

D. Inverted ΔTn*1999.2* *Ec*-HUCA 6, 7

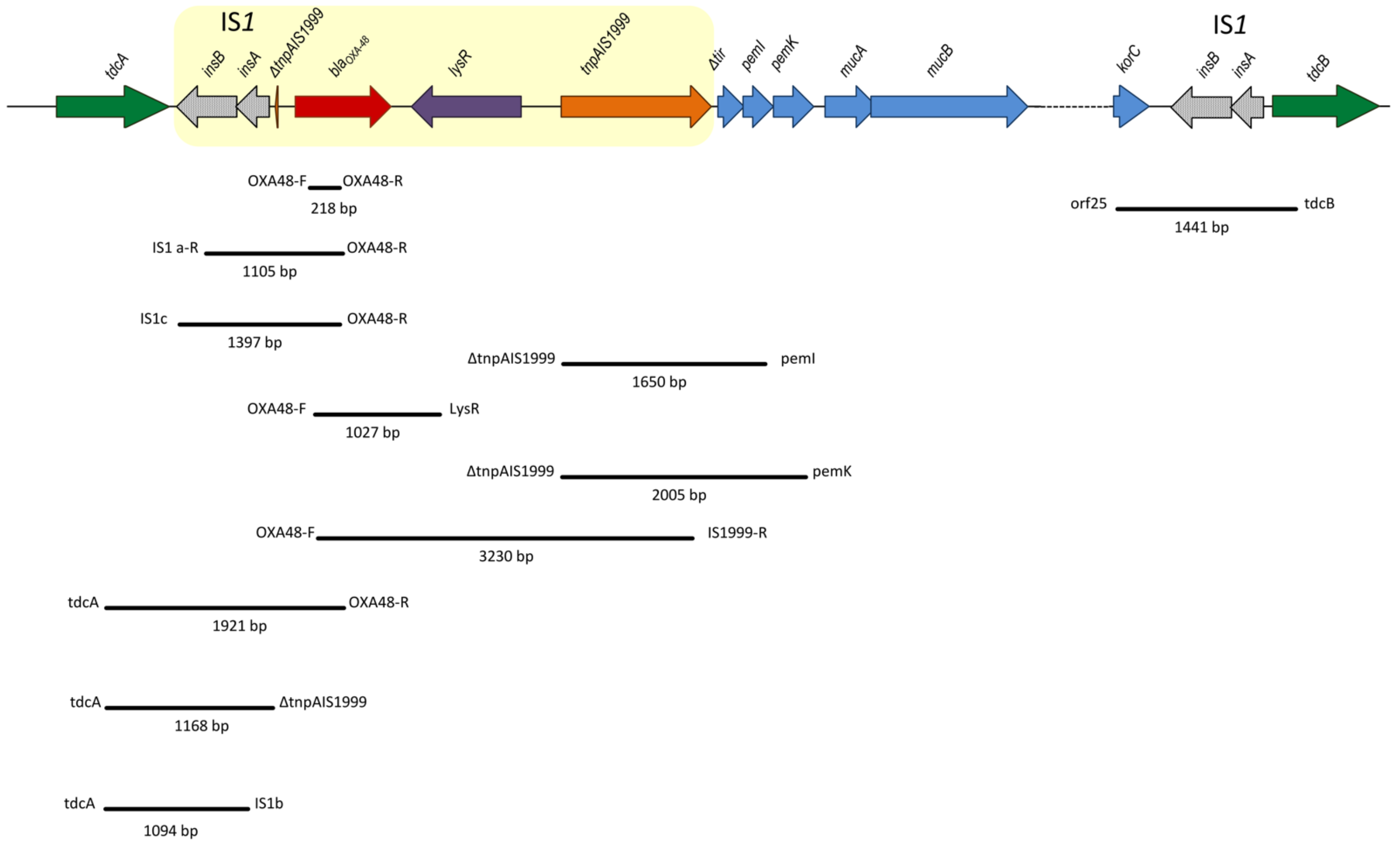

Supplementary Tables:

Table S1. Virulence genes detected by PCR in the seven OXA-48 *Escherichia coli* isolates

**Virulence genes**	***Ec*-HUCA 1**<br>O16:H5<br>B2<br>ST131<br>PST506<br>CH40-41	***Ec*-HUCA 2**<br>O9:H25<br>B1<br>ST58<br>PST24<br>CH4-27	***Ec*-HUCA 3**<br>O25:H5<br>B2<br>ST83<br>PST207<br>CH38-21	***Ec*-HUCA 4**<br>O25b:H4<br>B2<br>ST131<br>PST9<br>CH40-22	***Ec*-HUCA 5**<br>O25b:H4<br>B2<br>ST131<br>PST43<br>CH40-30	***Ec*-HUCA 6**<br>O25b:H4<br>B2<br>ST131<br>PST43<br>CH40-30	***Ec*-HUCA 7**<br>O25b:H4<br>B2<br>ST131<br>PST43<br>CH40-30
**Adhesins**							
fimC	+	+	+	+	+	+	+
fimH	+	+	+	+	+	+	+
fimAv$_{MT78}$	-	-	-	-	-	-	-
papAH	-	-	+	+	+	+	+
papC	-	-	+	+	+	+	+
papEF	-	-	+	+	+	+	+
papG I	-	-	-	-	-	-	-
papG II	-	-	-	-	+	+	+
papG III	-	-	+	+	-	-	-
sfa/focDE	-	-	-	-	-	-	-
afa/draBC	-	-	-	-	-	-	-
iha	+	-	-	+	+	+	+
matB	+	+	+	+	+	+	+
yfcV	-	-	+	+	+	+	+
**Toxins**							
cnf1	-	-	-	+	+	+	+
cdtB	-	-	+	-	-	-	-
sat	-	-	-	-	+	+	+
tsh	-	-	-	-	-	-	-
hlyA	-	-	-	+	+	+	+
hlyF	-	+	-	+	-	-	-
astA	-	-	-	-	-	-	-
pic	-	-	-	-	-	-	-
pet	+	-	+	+	+	+	+
vat	-	-	+	-	-	-	-
tosA	-	-	-	-	-	-	-
**Iron uptake**							
iucD	-	+	-	+	+	+	+
iutA	-	+	-	+	+	+	+
iroN	-	+	-	+	-	-	-
fyuA	+	+	+	+	+	+	+
chuA	+	-	+	+	+	+	+
ireA	-	-	-	-	-	-	-
irp2	+	+	+	+	+	+	+
sitA	+	+	+	+	+	+	+
hma	+	-	+	+	+	+	+
**Capsule**							
kii-kpsM II	+	-	+	+	+	+	+
kpsM II-K2	+	-	-	-	-	-	-
kpsM II-K5	-	-	+	+	+	+	+
neuC-K1	-	-	-	-	-	-	-
kpsM III	-	-	-	-	-	-	-
**Miscellaneous**							
cvaC	-	+	-	+	-	-	-
iss	-	+	-	+	-	-	-
traT	+	+	-	+	-	-	-
ibeA	-	-	+	+	-	-	-

malX (PAI)	+	-	+	+	+	+	+
usp	+	-	+	+	+	+	+
ompT	+	+	+	+	+	+	+
betA	+	+	+	+	+	+	+
hha	+	+	+	+	+	+	+
Hha2	+	-	-	+	+	+	+
Hha3	+	-	-	-	+	+	+
[a]ExPEC status	-	-	+	+	+	+	+
[b]UPEC status	-	+	+	+	+	+	+
Number of VG (n= 50)	20	16	24	33	29	29	29

[a]Strains presumptively regarded as extraintestinal pathogenic *E. coli* (ExPEC) since they were positive for two or more of five markers, including *papAH* and/or *papC*, *sfa/focDE*, *afa/draBC*, *kpsM II* and *iutA*[2,3].

[b]Strains presumptively regarded as uropathogenic *E. coli* (UPEC) because they were positive for three or more of four markers, including *chuA*, *fyuA*, *vat* and *yfcV*[3,4].

Table S2. Associations of molecular types, virulence-gene profiles and virulence patterns in the murine sepsis model of the 7 OXA-48 producing *Escherichia coli* isolates.

Isolate code	Serotype	Phylo-group	Virotype [a]	AST /PST [b]	Lethality [c] ≤24 h	Lethality [d] 25 h–48 h	Final [e] lethality	Lesion = Type – degree (No) [f]	VG score[g]	ExPEC status[h]
Ec-HUCA 1	O16:H5	B2	NT	131 / 506	0	0	0	Abscess ++ (8); necrosis + (2)	20	-
Ec-HUCA 2	O9:H25	B1	NA	58 / 24	0	0	1	Abscess +++(3); Abscess ++ (3); necrosis ++ (3)	16	-
Ec-HUCA 3	O25a:H5	B2	NA	83 /207	0	0	0	Abscess ++ (6); Abscess and necrosis ++ (4)	24	+
Ec-HUCA 4	O25b:H4	B2	D5	131 / 9	0	0	0	Necrosis +++ (8); necrosis ++++ (2)	33	+
Ec-HUCA 5	O25b:H4	B2	E	131 /43	0	0	0	Abscess and necrosis +++ (8); Abscess and necrosis ++++ (2)	29	+
Ec-HUCA 6	O25b:H4	B2	E	131 /43	1	0	1	Abscess and necrosis +++ (8); Abscess and necrosis ++++ (1)	29	+
Ec-HUCA 7	O25b:H4	B2	E	131 /43	0	0	1	Abscess and necrosis +++ (8); Abscess and necrosis ++++ (1)	29	+
CFT-073	O6:H1	B2	NA	73 / 4	4	6	10	None	*	+
K12-MG1655	O16:H48	A	NA	98 / 262	0	0	0	None	*	-

[a]Virotypes of the ST131 isolates established based on the presence or absence of 13 VF according to the scheme described by Dahbi *et al.*[1] Virulence profile of the O16:H5-ST131 isolate did not match any virotype of the scheme, so it was showed as NT (not typeable); NA (not applicable) for those non-ST131 isolates.

[b]Sequence types (STs) according to Achtman (AST) and Pasteur schemes (PST), respectively.

[c]Number of mice dead within 24 h postinjection.

[d]Number of mice dead within 25–48 h postinjection.

[e]Total number of mice dead within 7 days of experiment. For each isolate, 10 outbred female RjOrl:Swiss mice (3–4 weeks old; Janvier Labs, France) were assayed.

[f]Type and number of mice with acute inflammatory response in the neck region when euthanazized of those 10 assayed for each isolate. Type = abscess / necrosis; degree and extension = + to ++++.

[g]Virulence-gene (VG) score calculated as the sum of virulence genes detected out of the 50 analyzed by PCR per isolate; *For control strains, only a reduced number of virulence genes (23) was screened; as a result, CFT-073 was positive for *fimH papG II focG hlyA sat iutA iucD iroN kpsM II-K5 malX usp* and K12-MG1655 only for *fimH*.

[h]ExPEC status (+) defined as presence of two or more of five markers, including *papAH* and/or *papC*, *sfa/focDE*, *afa/draBC*, *kpsM II* and *iutA*[2,3].

Table S3.- SNP numbers are approximate averages of individual comparisons between each pair of genomes represented on Figure 4.

	AZ-TG-713-2	AZ-TG-WCHI-2	AZ-TG56421	BIDMC20B	BIDMC38	BWH24	CFSAN046659	E2022	E35BA	E61BA	EC958	Ec-HUCA1	Ec-HUCA2	Ec-HUCA3	Ec-HUCA4	Ec-HUCA5	Ec-HUCA6	Ec-HUCA7	ECOR-66	ESC0230	Eco889	Ecol_448	Ecol_743	FV9873	HVH177	JJ1886	JJ1887	JJ2434	KFu033	KKo004	MNCRE44	PNUSAE004413	SE15	ZH063	upec-58
**AZ-TG-713-2**		2940	3709	79276	79219	79276	3100	79268	79246	80784	79202	79715	4564	80923	80787	79247	79253	79253	80764	80758	79668	79666	79679	79214	80752	79195	79165	79206	2756	80843	79273	4426	80353	80663	81115
**AZ-TG-WCHI-2**	2940		2671	79335	79278	79335	1960	79327	79305	80710	79261	79569	3946	80806	80713	79306	79312	79312	80674	80668	79522	79520	79541	79273	80678	79254	79224	79266	1470	80763	79332	3885	80215	80583	80992
**AZ-TG56421**	3709	2671		79612	79555	79612	2601	79604	79582	80987	79538	79842	3511	81076	80990	79583	79589	79589	80980	80974	79795	79793	79806	79550	80955	79531	79501	79542	2461	81059	79609	3725	80480	80866	81273
**BIDMC20B**	79276	79335	79612		224	6	79632	122	229	3783	207	8341	79666	19809	3791	188	194	194	19999	19993	8350	8352	9008	219	3768	131	157	117	79470	20047	170	79708	8522	4026	19963
**BIDMC38**	79219	79278	79555	224		224	79575	220	233	3775	31	8210	79609	19742	3796	250	256	256	19932	19926	8219	8221	8942	43	3760	223	249	215	79413	19980	252	79651	8514	4122	19958
**BWH24**	79276	79335	79612	6	224		79632	122	229	3783	207	8341	79666	19809	3791	188	194	194	19999	19993	8350	8352	9008	219	3768	131	157	117	79470	20047	170	79708	8522	4026	19963
**CFSAN046659**	3100	1960	2601	79632	79575	79632		79624	79602	81007	79558	79851	3832	81096	81010	79603	79609	79609	80945	80939	79804	79802	79822	79570	80975	79551	79521	79562	1835	81025	79629	3953	80496	80879	81267
**E2022**	79268	79327	79604	122	220	122	79624		223	3775	203	8335	79658	19803	3785	182	188	188	19993	19987	8344	8346	9002	213	3762	125	151	103	79462	20041	164	79700	8516	4020	19957
**E35BA**	79246	79305	79582	229	233	229	79602	223		3745	216	8348	79636	19756	3751	233	239	239	19946	19940	8357	8359	8991	226	3732	230	256	218	79440	19994	238	79678	8486	4127	19924
**E61BA**	80784	80710	80987	3783	3775	3783	81007	3775	3745		3758	8017	81127	18093	192	3757	3763	3763	18283	18277	8026	8028	8701	3770	151	3752	3778	3772	80831	18330	3785	81169	6673	1016	17401
**EC958**	79202	79261	79538	207	31	207	79558	203	216	3758		8193	79592	19725	3779	233	239	239	19915	19909	8202	8204	8925	26	3743	206	232	198	79396	19963	235	79634	8497	4105	19941
**Ec-HUCA1**	79715	79569	79842	8341	8210	8341	79851	8335	8348	8017	8193		79869	19554	8038	8331	8337	8337	19962	19956	93	93	1317	8205	8002	8262	8288	8330	79744	19650	8347	79889	1704	8325	19848
**Ec-HUCA2**	4564	3946	3511	79666	79609	79666	3832	79658	79636	81127	79592	79869		81228	81128	79637	79643	79643	81199	81193	79856	79854	79867	79604	81093	79585	79589	79630	3660	81239	79663	1466	80541	81038	81485
**Ec-HUCA3**	80923	80806	81076	19809	19742	19809	81096	19803	19756	18093	19725	19554	81228		18076	19784	19790	19790	1034	1040	19543	19543	19996	19737	18058	19767	19773	19778	80962	847	19816	81260	19705	18211	2704
**Ec-HUCA4**	80787	80713	80990	3791	3796	3791	81010	3785	3751	192	3779	8038	81128	18076		3752	3758	3758	18266	18260	8047	8049	8725	3791	299	3760	3786	3780	80834	18313	3785	81170	6821	1024	17539
**Ec-HUCA5**	79247	79306	79583	188	250	188	79603	182	233	3757	233	8331	79637	19784	3752		14	14	19973	19967	8340	8342	8979	245	3742	187	213	177	79441	20023	198	79679	8500	4036	19934
**Ec-HUCA6**	79253	79312	79589	194	256	194	79609	188	239	3763	239	8337	79643	19790	3758	14		2	19979	19973	8346	8348	8985	251	3748	193	219	183	79447	20029	204	79685	8506	4042	19940
**Ec-HUCA7**	79253	79312	79589	194	256	194	79609	188	239	3763	239	8337	79643	19790	3758	14	2		19979	19973	8346	8348	8985	251	3748	193	219	183	79447	20029	204	79685	8506	4042	19940
**ECOR-66**	80764	80674	80980	19999	19932	19999	80945	19993	19946	18283	19915	19962	81199	1034	18266	19973	19979	19979		8	19915	19915	20368	19927	18248	19957	19927	19932	80877	1217	20006	81189	20077	18366	3030
**ESC0230**	80758	80668	80974	19993	19926	19993	80939	19987	19940	18277	19909	19956	81193	1040	18260	19967	19973	19973	8		19909	19909	20362	19921	18242	19951	19921	19926	80871	1223	20000	81183	20071	18360	3036
**Eco889**	79668	79522	79795	8350	8219	8350	79804	8344	8357	8026	8202	93	79856	19543	8047	8340	8346	8346	19915	19909		48	1262	8214	7991	8271	8241	8283	79697	19603	8355	79842	1657	8278	19801
**Ecol_448**	79666	79520	79793	8352	8221	8352	79802	8346	8359	8028	8204	93	79854	19543	8049	8342	8348	8348	19915	19909	48		1272	8216	7991	8273	8243	8285	79695	19603	8358	79840	1657	8278	19801
**Ecol_743**	79679	79541	79806	9008	8942	9008	79822	9002	8991	8701	8925	1317	79867	19996	8725	8979	8985	8985	20368	20362	1262	1272		8937	8666	8953	8923	8941	79708	20056	9012	79853	2322	8987	20367
**FV9873**	79214	79273	79550	219	43	219	79570	213	226	3770	26	8205	79604	19737	3791	245	251	251	19927	19921	8214	8216	8937		3755	218	244	210	79408	19975	247	79646	8509	4117	19953
**HVH177**	80752	80678	80955	3768	3760	3768	80975	3762	3732	151	3743	8002	81093	18058	299	3742	3748	3748	18248	18242	7991	7991	8666	3755		3737	3743	3737	80805	18295	3770	81135	6636	979	17366
**JJ1886**	79195	79254	79531	131	223	131	79551	125	230	3752	206	8262	79585	19767	3760	187	193	193	19957	19951	8271	8273	8953	218	3737		30	120	79389	20005	169	79627	8491	3997	19932
**JJ1887**	79165	79224	79501	157	249	157	79521	151	256	3778	232	8288	79589	19773	3786	213	219	219	19927	19921	8241	8243	8923	244	3743	30		90	79359	19975	195	79597	8461	3967	19902
**JJ2434**	79206	79266	79542	117	215	117	79562	103	218	3772	198	8330	79630	19778	3780	177	183	183	19932	19926	8283	8285	8941	210	3737	120	90		79400	19980	159	79638	8455	3959	19896
**KFu033**	2756	1470	2461	79470	79413	79470	1835	79462	79440	80831	79396	79744	3660	80962	80834	79441	79447	79447	80877	80871	79697	79695	79708	79408	80805	79389	79359	79400		80920	79467	3620	80382	80724	81166
**KKo004**	80843	80763	81059	20047	19980	20047	81025	20041	19994	18330	19963	19650	81239	847	18313	20023	20029	20029	1217	1223	19603	19603	20056	19975	18295	20005	19975	19980	80920		20054	81227	19765	18415	2610
**MNCRE44**	79273	79332	79609	170	252	170	79629	164	238	3785	235	8347	79663	19816	3785	198	204	204	20006	20000	8355	8358	9012	247	3770	169	195	159	79467	20054		79705	8524	4048	19963
**PNUSAE004413**	4426	3885	3725	79708	79651	79708	3953	79700	79678	81169	79634	79889	1466	81260	81170	79679	79685	79685	81189	81183	79842	79840	79853	79646	81135	79627	79597	79638	3620	81227	79705		80527	81046	81437
**SE15**	80353	80215	80480	8522	8514	8522	80496	8516	8486	6673	8497	1704	80541	19705	6821	8500	8506	8506	20077	20071	1657	1657	2322	8509	6636	8491	8461	8455	80382	19765	8524	80527		7407	18791
**ZH063**	80663	80583	80866	4026	4122	4026	80879	4020	4127	1016	4105	8325	81038	18211	1024	4036	4042	4042	18366	18360	8278	8278	8987	4117	979	3997	3967	3959	80724	18415	4048	81046	7407		17747
**upec-58**	81115	80992	81273	19963	19958	19963	81267	19957	19924	17401	19941	19848	81485	2704	17539	19934	19940	19940	3030	3036	19801	19801	20367	19953	17366	19932	19902	19896	81166	2610	19963	81437	18791	17747	

Table S4. Primers used to establish the genetic context of the *bla*$_{OXA-48}$ gene.

Primer name	Sequence (5'-3')	Reference
MultiOXA-48_fo	GCTTGATCGCCCTCGATT	5
MultiOXA-48_rev	GATTTGCTCCGTGGCCGAAA	5
IS1999-R	CAGCAATTCTTTCTCCGTG	6
ΔtnpAIS1999	ATGCGCGAAGCCAATATCCTAC	This study
LysR	GCTAGTGCCAATCTTACAGG	6
IS1a-R	GTAAAACAGCCAGCGCTGGCGCG	7
IS1 b	TGAGGTGCTCCAGTGGCTTC	This study
IS1 c	GCCCGATGACTTTGTCATGC	This study
pemI	TGACCATGCCAACTTCATT	6
orf25	GGAAATAGCAGCGCAAAA	6
orf67	CAGCCAGAATAAGAGCAATC	6
tdcA	AGAACGAACAGTGGGTGTTG	This study
tdcB	TTCCGCATCAGTCAGTGAAC	This study
morA	GATGGGCATCTGCAATACTC	This study

Table S5. Primers used in this study for PCR amplification of virulence genes

Gene	Function	Primer name	Sequence (5'-3')	Reference
**Adhesins**				
fimC	D-mannose-specific adhesion, type 1 fimbriae	fimC-F<br>fimC-R	TAAGGAAATCGCAGGAA<br>GTCAGGTAATAGGGTGTCG	8
fimH	D-mannose-specific adhesion, type 1 fimbriae	fimH-F<br>fimH-R	TGCAGAACGGATAAGCCGTGG<br>GCAGTCACCTGCCCTCCGGTA	9
fimAv $_{MT78}$	Fim A variant MT78 of type 1 fimbriae	fimA215<br>fimA201	ACTTTAGGATGAGTACTG<br>TCTGGCTGATACTACACC	10
papAH	P fimbriae (pyelonephritis-associated pilus) operon. Major structural subunit. Defines F antigen	papAH-F<br>papAH-R	ATGGCAGTGGTGTCTTTTGGTG<br>CGTCCCACCATACGTGCTCTTC	2
papC	P fimbriae operon. Pilus assembly	papC-F<br>papC-R	GTGGCAGTATGAGTAATGACCGTTA<br>ATATCCTTTCTGCAGGGATGCAATA	2
papEF	P fimbriae operon. Minor tip pilins. Connect PapG to PapA	papEF-F<br>papEF-R	GCAACAGCAACGCTGGTTGCATCAT<br>AGAGAGAGCCACTCTTATACGGACA	11
papG I	P fimbriae operon. Allele I of *papG* gene	Pap-I f<br>Pap-I r	TTAGCTGGATGGCACAATG<br>TTGTCCATGTATCCCATTCAT	12
papG II	P fimbriae operon. Allele II of *papG* gene	Pap-II f<br>Pap-II r	GGGCATTGCTACGGTAACCTG<br>CGCTATTAATAGACAGATCACC	12
papG III	P fimbriae operon. Allele III of *papG* gene	Pap-III f<br>Pap-III r	CGGCAACTTTAAGCTATGTG<br>TGTACCATCTCATCGTTGTCTC	12
sfa/focDE	Central region of *sfa* (S fimbriae) and *foc* (F1C fimbriae) operons	sfa1<br>sfa2	CTCCGGAGAACTGGGTGCATCTTAC<br>CGGAGGAGTAATTACAAACCTGGCA	13
afa/draBC	Dr antigen-specific adhesion operons (AFA, Dr, F1845)	afa1<br>afa2	GCTGGGCAGCAAACTGATAACTCTC<br>CATCAAGCTGTTTGTTCGTCCGCCG	13
iha	Nonhemagglutinin adhesion (from O157:H7 and CFT073)	iha-F<br>iha-R	CTGGCGGAGGCTCTGAGATCA<br>TCCTTAAGCTCCCGCGGCTGA	2

matB	Meningitis and temperature-dependent fimbriae	matB-F<br>matB-R	TTAACTGGTCCAGGTCGCGT<br>ATGAAAAAAAAGGTTCTGGCA	14
yfcV	Putative chaperone-usher fimbria	yfcV forward<br>yfcV reverse	ACATGGAGACCACGTTCACC<br>GTAATCTGGAATGTGGTCAGG	15
**Toxins**				
cnf1	Cytotoxic necrotizing factor 1	cnf1-f2<br>cnf1-rc	CAGGAGGTACTTAGCAGCGT<br>TAATTTTGGGTTTGTATC	12
cdtB	Cytolethal distending toxin	cdt-s1<br>cdt-as1<br>cdt-s2<br>cdt-as2	GAAAGTAAATGGAATATAAATGTCCG<br>AAATCACCAAGAATCATCCAGTTA<br>GAAAATAAATGGAACACACATGTCCG<br>AAATCTCCTGCAATCATCCAGTTA	16
sat	Secreted autotransporter toxin	satF<br>satR	GCAGCTACCGCAATAGGAGGT<br>CATTCAGAGTACCGGGGCCTA	17
tsh	Temperature-sensitive hemagglutinin. Serine protease	tsh03<br>tsh15	GGTGGTGCACTGGAGTGG<br>AGTCCAGCGTGATAGTGG	18
hlyA	α-hemolysin	hly f<br>hly r	AACAAGGATAAGCACTGTTCTGGCT<br>ACCATATAAGCGGTCATTCCCGTCA	11
hlyF	Hemolysin F	hlyF f<br>hlyF r	TCGTTTAGGGTGCTTACCTTCAAC<br>TTTGGCGGTTTAGGCATTCC	19
astA	(EAST 1) enteroaggregative *E. coli* heat stable toxin	astA forward<br>astA reverse	CCATCAACACAGTATATCCG<br>GGTCGCGAGTGACGGCTTTG	2
pic	Protein involved in intestinal colonization. Serine protease	pic forward<br>pic reverse	GGGTATTGTCCGTTCCGAT<br>ACAACGATACCGTCTCCCG	2
pet	Plasmid-encoded toxin	pet forward<br>pet reverse	GACCATGACCTATACCGACAGC<br>CCGATTTCTCAAACTCAAGACC	20
vat	Vacuolating autotransporter toxin. Serine protease	vat forward<br>vat reverse	TCAGGACACGTTCAGGCATTCAGT<br>GGCCAGAACATTTGCTCCCTTGTT	15
tosA	In vivo-induced repeat-in toxin (RTX) family member	tosA forward<br>tosA reverse	TGGGCATTCTGATGCTGCTGACTA<br>TTGTTCCGTCTGGCCCATTATCCT	21
**Iron uptake**				

iucD	Aerobactin	Aer f<br>Aer r	TACCGGATTGTCATATGCAGACCGT<br>AATATCTTCCTCCAGTCCGGAGAAG	11
iutA	Ferric aerobactin receptor (iron uptake transport)	aer-851f<br>aer-1152r	GGCTGGACATCATGGGAACTGG<br>CGTCGGGAACGGGTAGAATCG	9
iroN	Catecholate (salmochelin) siderophore receptor	Ironec-f<br>Ironec-r	AAGTCAAAGCAGGGGTTGCCCG<br>GACGCCGACATTAAGACGCAG	9
fyuA	*Yersinia* siderophore receptor (ferric yersiniabactin uptake)	fyuA forward<br>fyuA reverse	GTAAACAATCTTCCCGCTCGGCAT<br>TGACGATTAACGAACCGGAAGGGA	15
chuA	Heme binding outer membrane	chuA-ITU-F<br>chuA-ITU-R	CTGAAACCATGACCGTTACG<br>TTGTAGTAACGCACTAAACC	15
ireA	Iron-regulated element (siderophore receptor)	ireA-F<br>ireA-R	GATGACTCAGCCACGGGTAA<br>CCAGGACTCACCTCACGAAT	2
irp2	Iron-represible protein (In HPI)	irp2-F<br>irp2-R	AAGGATTCGCTGTTACCGGAC<br>TCGTCGGGCAGCGTTTCTTCT	22
sitA	Iron uptake system	sitA-F<br>sitA-R	AGGGGGCACAACTGATTCTCG<br>TACCGCGCCGTTTTCTGTGC	23
hma	Heme uptake	hma-F<br>hma-R	ACATTGCAGAACTCCACCAATGCC<br>TTGATTCTTCCCAGCCTGCTGTTG	21
**Capsule**				
kii-kpsM II	Group II capsule	KpsII f<br>KpsII r	GCGCATTTGCTGATACTGTTG<br>CATCCAGACGATAAGCATGAGCA	9
kpsM II-K2	K2 variant of group II capsule	kpsII f<br>KpsII-K2r	GCGCATTTGCTGATACTGTTG<br>AGGTAGTTCAGACTCACACCT	24
kpsM II-K5	K5 variant of group II capsule	K5 f<br>kpsII r	CAGTATCAGCAATCGTTCTGTA<br>CATCCAGACGATAAGCATGAGCA	9
neuC-K1	K1 variant of group II capsule	neu1<br>neu2	AGGTGAAAAGCCTGGTAGTGTG<br>GGTGGTACATCCCGGGATGTC	25
kpsM III	Group III capsule	kps III f<br>kps III r	TCCTCTTGCTACTATTCCCCCT<br>AGGCGTATCCATCCCTCCTAAC	9

**Miscellaneous**				
cvaC	ColV (microcin V); on plasmids with *iucD/iutA, iroN, sitA*, *iss* and *traT*	ColV-Cf<br>ColV-Cr	CACACACAAACGGGAGCTGTT<br>CTTCCCGCAGCATAGTTCCAT	9
iss	Increased serum survival (outer membrane protein)	is-f<br>is-r	CAGCAACCCGAACCACTTGATG<br>AGCATTGCCAGAGCGGCAGAA	26
traT	Serum resistance-associated (outer membrane protein)	TraT f<br>TraT r	GGTGTGGTGCGATGAGCACAG<br>CACGGTTCAGCCATCCCTGAG	9
ibeA	Invasion of brain endothelium	ibe10 f<br>ibe10 r	AGGCAGGTGTGCGCCGCGTAC<br>TGGTGCTCCGGCAAACCATGC	9
***malX* (PAI)**	Pathogenicity-associated island marker (PAI)	MALX-F<br>MALX-R	GCATGAGCAGTGCGATACATCGC<br>AGGGCTGGGAAGTGGTTTAGCC	12
usp	Uropathogenic-specific protein (bacteriocin)	usp-f<br>usp-r	ACATTCACGGCAAGCCTCAG<br>AGCGAGTTCCTGGTGAAAGC	27
ompT	Outer membrane protein (protease) T	ompT-F<br>ompT-R	ATCTAGCCGAAGAAGGAGGC<br>CCCGGGTCATAGTGTTCATC	2
betA	Choline dehydrogenase	betA-F<br>betA-R	CGGTTTCGGCGTGGTTAC<br>GTGGGCATGTGCGGATTA	8
hha	Gene regulator in the enterobacteria	hha forward/<br>hha reverse	GTTTACGTCGTTGCAGACAA/<br>TTCCATACTGAGGAAGGGATCT	20
hha2	Gene regulator in the enterobacteria	4516 forward/<br>4516 reverse	CTTACCCTTGCGGAGCTGGA/<br>TATTTCCAGACAGAAGCCGGAA	20
hha3	Gene regulator in the enterobacteria	4796 forward/<br>4796 reverse	GAGACGCGGGAAAAGTGAAT/<br>CAGGTGACATATTTCCAGACGG	20

Supplementary References

1. Kado, C. I. & Liu, S. T. Rapid procedure for detection and isolation of large and small plasmids. *J. Bacteriol.* **145,** 1365–73 (1981).

2. Johnson, J. R. *et al.* Host Characteristics and Bacterial Traits Predict Experimental Virulence for Escherichia coli Bloodstream Isolates From Patients With Urosepsis. *Open forum Infect. Dis.* **2,** ofv083 (2015).

3. Johnson, J. R. *et al.* Isolation and molecular characterization of nalidixic acid-resistant extraintestinal pathogenic Escherichia coli from retail chicken products. *Antimicrob. Agents Chemother.* **47,** 2161–8 (2003).

4. Spurbeck, R. R. *et al.* Escherichia coli isolates that carry vat, fyuA, chuA, and yfcV efficiently colonize the urinary tract. *Infect. Immun.* **80,** 4115–22 (2012).

5. Dallenne, C., Da Costa, A., Decré, D., Favier, C. & Arlet, G. Development of a set of multiplex PCR assays for the detection of genes encoding important beta-lactamases in Enterobacteriaceae. *J. Antimicrob. Chemother.* **65,** 490–5 (2010).

6. Beyrouthy, R. *et al.* IS1R-mediated plasticity of IncL/M plasmids leads to the insertion of bla OXA-48 into the Escherichia coli Chromosome. *Antimicrob. Agents Chemother.* **58,** 3785–90 (2014).

7. Rodríguez, I., Rodicio, M. R., Mendoza, M. C. & Cruz Martín, M. Large conjugative plasmids from clinical strains of Salmonella enterica serovar virchow contain a class 2 integron in addition to class 1 integrons and several non-integron-associated drug resistance determinants. *Antimicrob. Agents Chemother.* **50,** 1603–7 (2006).

8. Wang, Y., Tang, C., Yu, X., Xia, M. & Yue, H. Distribution of serotypes and virulence-associated genes in pathogenic Escherichia coli isolated from ducks. *Avian Pathol.* **39,** 297–302 (2010).

9. Johnson, J. R. & Stell, A. L. Extended virulence genotypes of Escherichia coli strains from patients with urosepsis in relation to phylogeny and host compromise. *J. Infect. Dis.* **181,** 261–72 (2000).

10. Marc, D. & Dho-Moulin, M. Analysis of the fim cluster of an avian O2 strain of Escherichia coli: serogroup-specific sites within fimA and nucleotide sequence of fimI. *J. Med. Microbiol.* **44,** 444–52 (1996).

11. Yamamoto, S. *et al.* Detection of urovirulence factors in Escherichia coli by multiplex polymerase chain reaction. *FEMS Immunol. Med. Microbiol.* **12,** 85–90 (1995).

12. Mora, A. *et al.* Poultry as reservoir for extraintestinal pathogenic Escherichia coli O45:K1:H7-B2-ST95 in humans. *Vet. Microbiol.* **167,** 506–12 (2013).

13. Le Bouguenec, C., Archambaud, M. & Labigne, A. Rapid and specific detection of the pap, afa, and sfa adhesin-encoding operons in uropathogenic Escherichia coli strains by polymerase chain reaction. *J. Clin. Microbiol.* **30,** 1189–93 (1992).

14. Lehti, T. A., Heikkinen, J., Korhonen, T. K. & Westerlund-Wikström, B. The response regulator RcsB activates expression of Mat fimbriae in meningitic Escherichia coli. *J. Bacteriol.* **194,** 3475–85 (2012).

15. Spurbeck, R. R. *et al.* Escherichia coli Isolates That Carry vat, fyuA, chuA, and yfcV Efficiently Colonize the Urinary Tract. *Infect. Immun.* **80,** 4115–4122 (2012).

16. Tóth, I., Hérault, F., Beutin, L. & Oswald, E. Production of cytolethal distending toxins by pathogenic Escherichia coli strains isolated from human and animal sources: establishment of the existence of a new cdt variant (Type IV). *J. Clin. Microbiol.* **41,** 4285–91 (2003).

17. Johnson, J. R., Gajewski, A., Lesse, A. J. & Russo, T. A. Extraintestinal pathogenic Escherichia coli as a cause of invasive nonurinary infections. *J. Clin. Microbiol.* **41,** 5798–802 (2003).

18. Dozois, C. M. *et al.* Relationship between the Tsh autotransporter and pathogenicity of avian Escherichia coli and localization and analysis of the Tsh genetic region. *Infect. Immun.* **68,** 4145–54 (2000).

19. Morales, C., Lee, M. D., Hofacre, C. & Maurer, J. J. Detection of a novel virulence gene and a Salmonella virulence homologue among Escherichia coli isolated from broiler chickens. *Foodborne Pathog. Dis.* **1,** 160–5 (2004).

20. Prieto, A. *et al.* Tracking bacterial virulence: global modulators as indicators. *Sci. Rep.* **6,** 25973 (2016).

21. Vigil, P. D. *et al.* Presence of putative repeat-in-toxin gene tosA in Escherichia coli predicts successful colonization of the urinary tract. *MBio* **2,** e00066-11 (2011).

22. Schubert, S., Rakin, A., Karch, H., Carniel, E. & Heesemann, J. Prevalence of the "high-pathogenicity island" of Yersinia species among Escherichia coli strains that are pathogenic to humans. *Infect. Immun.* **66,** 480–5 (1998).

23. Runyen-Janecky, L. J., Reeves, S. A., Gonzales, E. G. & Payne, S. M. Contribution of the Shigella flexneri Sit, Iuc, and Feo iron acquisition systems to iron acquisition in vitro and in cultured cells. *Infect. Immun.* **71,** 1919–28 (2003).

24. Johnson, J. R. & O'Bryan, T. T. Detection of the Escherichia coli group 2 polysaccharide capsule synthesis Gene kpsM by a rapid and specific PCR-based assay. *J. Clin. Microbiol.* **42,** 1773–6 (2004).

25. Moulin-Schouleur, M. *et al.* Common virulence factors and genetic relationships between O18:K1:H7 Escherichia coli isolates of human and avian origin. *J. Clin. Microbiol.* **44,** 3484–92 (2006).

26. Rodriguez-Siek, K. E., Giddings, C. W., Doetkott, C., Johnson, T. J. & Nolan, L. K. Characterizing the APEC pathotype. *Vet. Res.* **36,** 241–56 (2005).

27. Bauer, R. J. *et al.* Molecular Epidemiology of 3 Putative Virulence Genes for *Escherichia coli* Urinary Tract Infection— *usp, iha,* and *iroN* $_{E.\ coli}$. *J. Infect. Dis.* **185,** 1521–1524 (2002).
